# Supplementary material for: Genome-wide chromosome architecture prediction reveals biophysical principles underlying gene structure
Source: Cell Genom. 2024 Nov 25;4(12):100698. doi: 10.1016/j.xgen.2024.100698 (PMC11701261; doi:10.1016/j.xgen.2024.100698)
Supplement: Document S1. Figures S1–S4 [file mmc1.pdf]

Cell Genomics, Volume *4*

Supplemental information

Genome-wide chromosome architecture prediction reveals biophysical principles underlying gene structure

Michael Chiang, Chris A. Brackley, Catherine Naughton, Ryu-Suke Nozawa, Cleis Battaglia, Davide Marenduzzo, and Nick Gilbert

SUPPLEMENTAL FIGURES

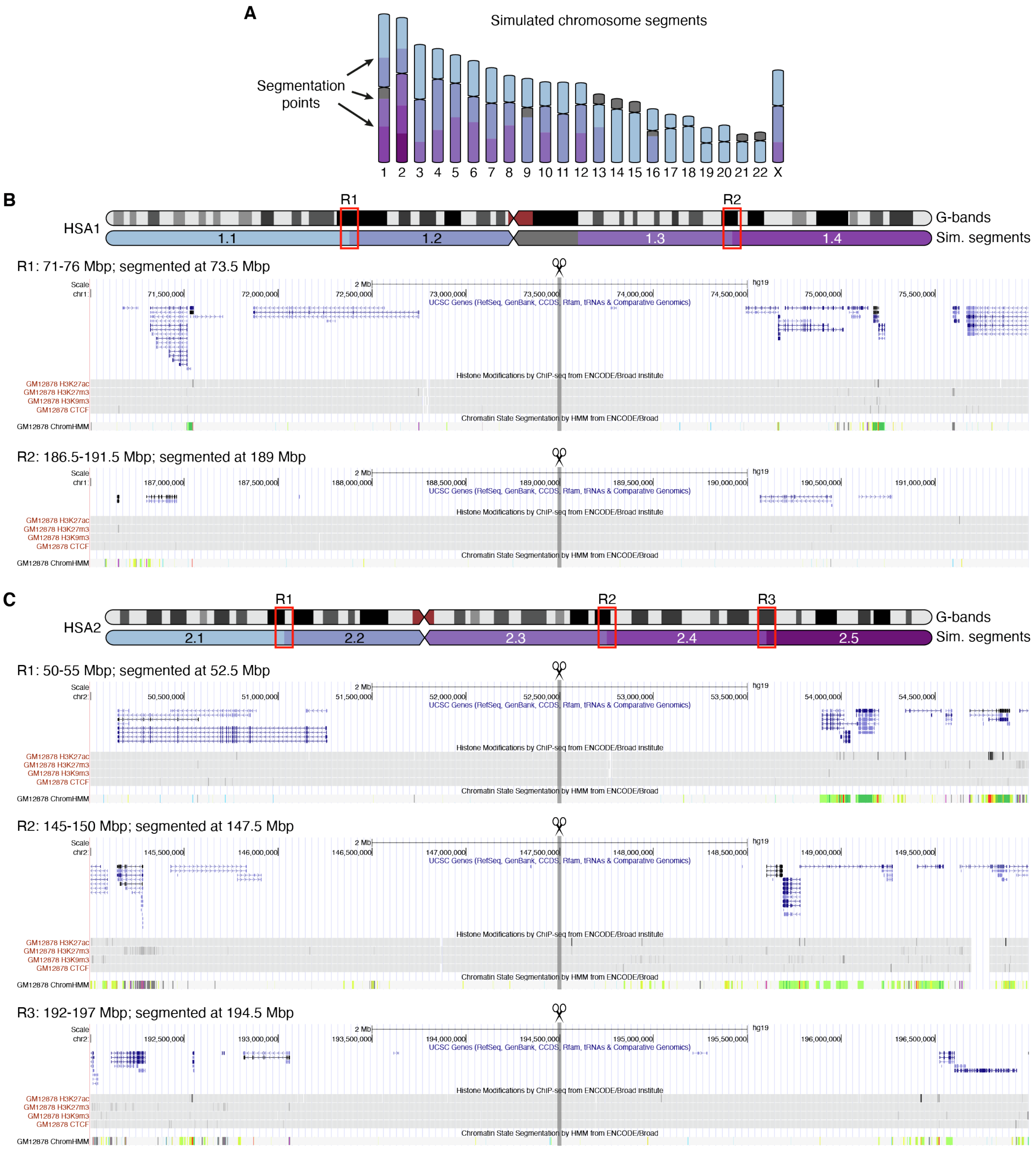

Figure S1. Long chromosomes were divided into shorter fragments when performing e-HiP-HoP simulations, related to Figure 1 and STAR Methods.

(A) Ideograms showing the segmentation points within each chromosome.

(B and C) Simulated chromosome fragments in (B) HSA1 and (C) HSA2 and the chromatin context near segmentation points. Ideograms at the top indicate the positions of segmentation (the region colored in gray is unmappable within the hg19 reference genome and was not simulated). Snapshots from the UCSC genome browser (bottom) show the genes, various ChIP-seq signals (H3K27ac, H3K27me3, H3K9me3, and CTCF), and the 15 chromatin-state hidden Markov model (ChromHMM) track within a 5 Mbp region around segmentation points. These tracks demonstrate that there are no notable genomic and epigenomic features near the segmentation points.

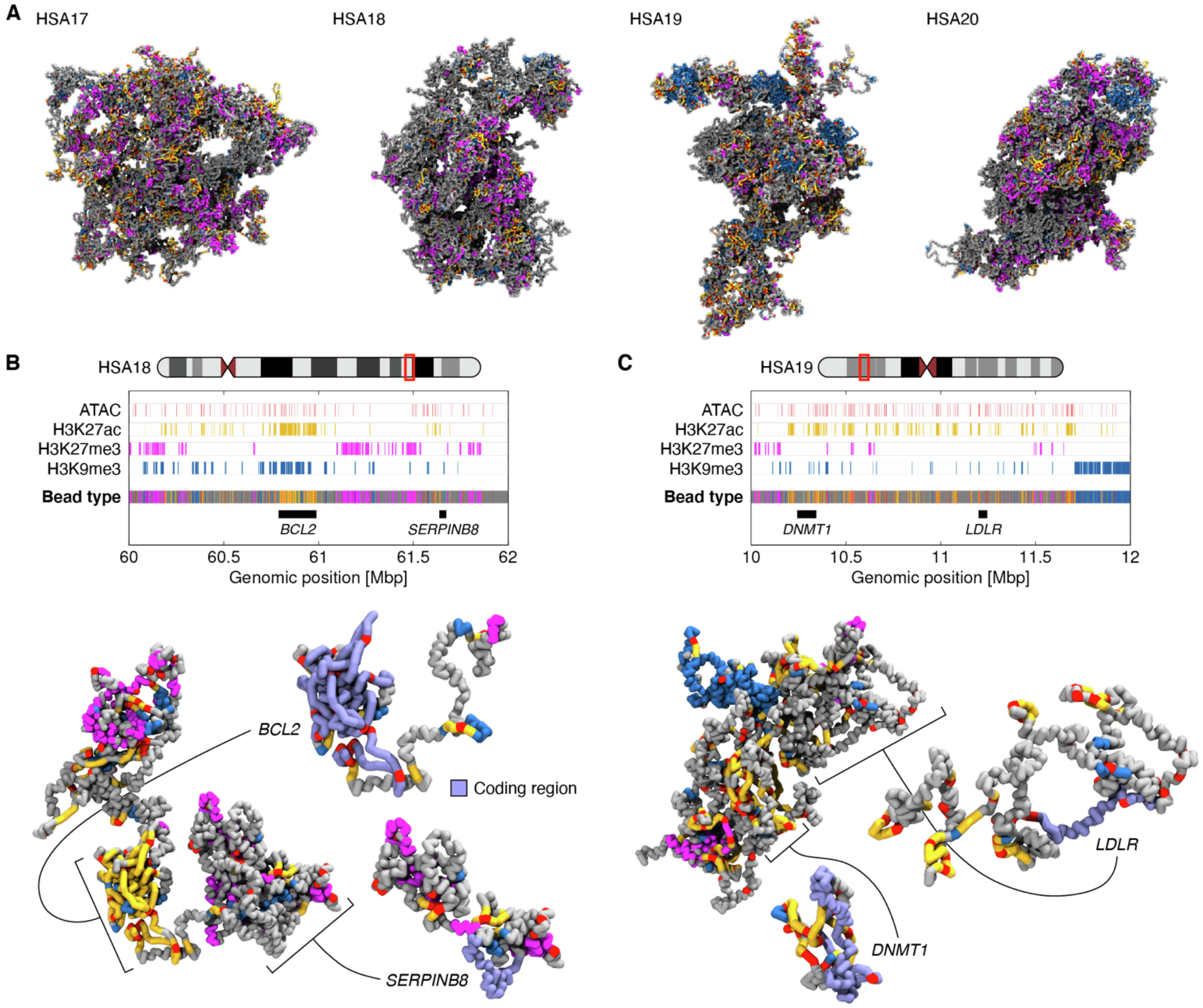

Figure S2. Simulated structures from e-HiP-HoP provide a detailed view of the 3D conformation of individual genes and their neighborhood, related to Figure 1.

(A) Representative snapshots of the predicted structures of HSA17, 18, 19, and 20, all of which were simulated individually as a single, continuous polymer without segmentation.

(B) An enlarged view of HSA18 at 60–62 Mbp. Epigenetic tracks used as model inputs and the resulting bead type along the chromatin fiber are shown at the top, and an example structure of this region is shown at the bottom, with a further expanded view of two gene topoi, *BCL2* and *SERPINB8* (their coding region is colored in light purple).

(C) Similar to (B) but for the region 10–12 Mbp in HSA19, with the conformations of the topoi of genes *DNMT1* and *LDLR* shown.

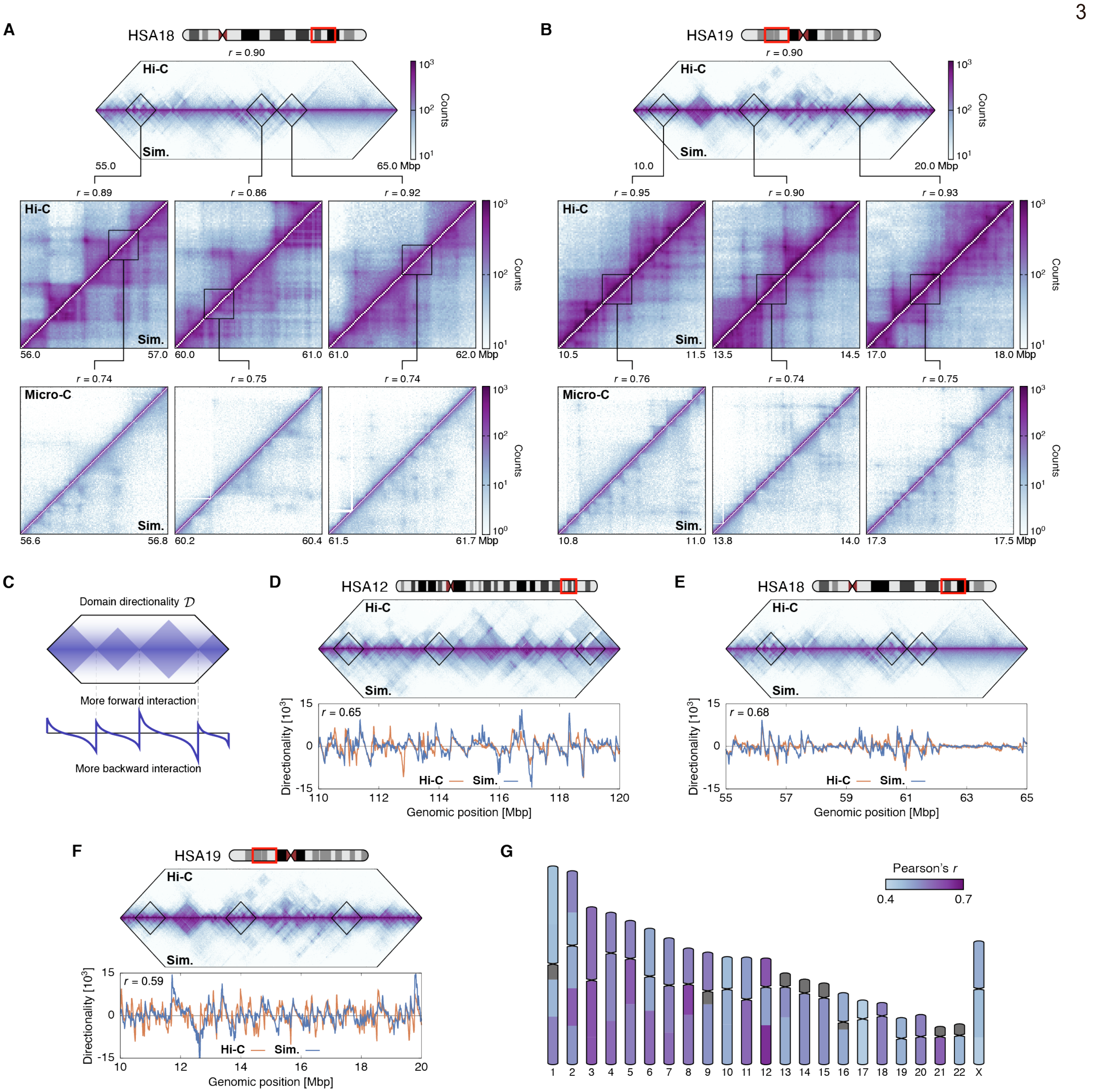

Figure S3. Comparison of contact maps between Hi-C, Micro-C, and e-HiP-HoP simulations, related to Figure 2.

(A and B) Comparison of contact maps between Hi-C, Micro-C, and e-HiP-HoP simulations for the regions (A) HSA18:55–65 Mbp and (B) HSA19:10–20 Mbp. The top panel shows the comparison of the full region between Hi-C and simulations at 10 kbp resolution, with enlarged views of the 1 Mbp boxed regions shown in the middle panel. The bottom panel compares the maps between Micro-C and simulations at 1 kbp resolution for the 200 kbp boxed regions marked in the middle panel. Pearson correlation coefficient r is reported for each comparison, and all correlations are statistically significant with $p < 10^{-10}$.

(C) A schematic explaining the directionality score D which quantitatively captures domains seen in contact maps. The D score at a chromatin bin is the total contact frequencies of the bin with other bins to its left, up to a threshold, minus the total contact frequencies with those to its right (see STAR Methods).

(D–F) Comparing the directionality profiles of the contact maps between Hi-C and simulations for (D) the region HSA12:110–120 Mbp (as examined in Figure 2A) and the two regions shown above, (E)

HSA18:55–65 Mbp and (F) HSA19:10–20 Mbp. Here, the top panel shows the comparison of the contact maps at 10 kbp resolution, and the bottom panel plots the directionality profiles. Pearson correlation coefficient *r* is reported for comparing these profiles and is statistically significant ($p < 10^{-10}$) for all the regions shown here.

(G) Ideograms showing the Pearson correlation coefficient between the directionality profiles from Hi-C and simulated contact maps for each chromosome segment (gray regions were not simulated as they are unmappable regions within the reference genome).

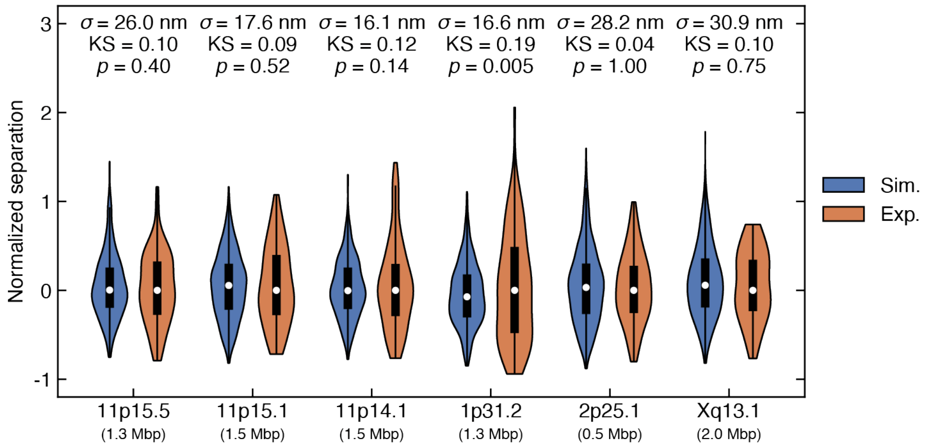

Figure S4. Further validation of e-HiP-HoP simulations with FISH data, related to Figure 2. Comparing the simulated and experimental FISH distance distributions for six pairs of probes. As in Figure 2C, we tested whether the difference between the distributions is statistically significant using the two-sample Kolmogorov-Smirnov (KS) test (see STAR Methods). Of all, the simulated and experimental distributions only show significant deviation for the probe pair within 1p31.2, which is a gene-poor region and is therefore less relevant to our analysis on actively transcribed chromatin. Experimental data were taken from ref. S1.

Supplemental References

[S1] Nozawa, R.S., Boteva, L., Soares, D.C., Naughton, C., Dun, A.R., Buckle, A., Ramsahoye, B., Bruton, P.C., Saleeb, R.S., Arnedo, M., et al. (2017). SAF-A Regulates Interphase Chromosome Structure through Oligomerization with Chromatin-Associated RNAs. Cell *169*, 1214–1227.e18. https://doi.org/10.1016/j.cell.2017.05.029.
