# Supplementary material for: Genome-wide chromosome architecture prediction reveals biophysical principles underlying gene structure
Source: Cell Genom. 2024 Nov 25;4(12):100698. doi: 10.1016/j.xgen.2024.100698 (PMC11701261; doi:10.1016/j.xgen.2024.100698)
Supplement: Document S2. Article plus supplemental information [file mmc2.pdf]

Cell Genomics

Article

Genome-wide chromosome architecture prediction reveals biophysical principles underlying gene structure

Graphical abstract

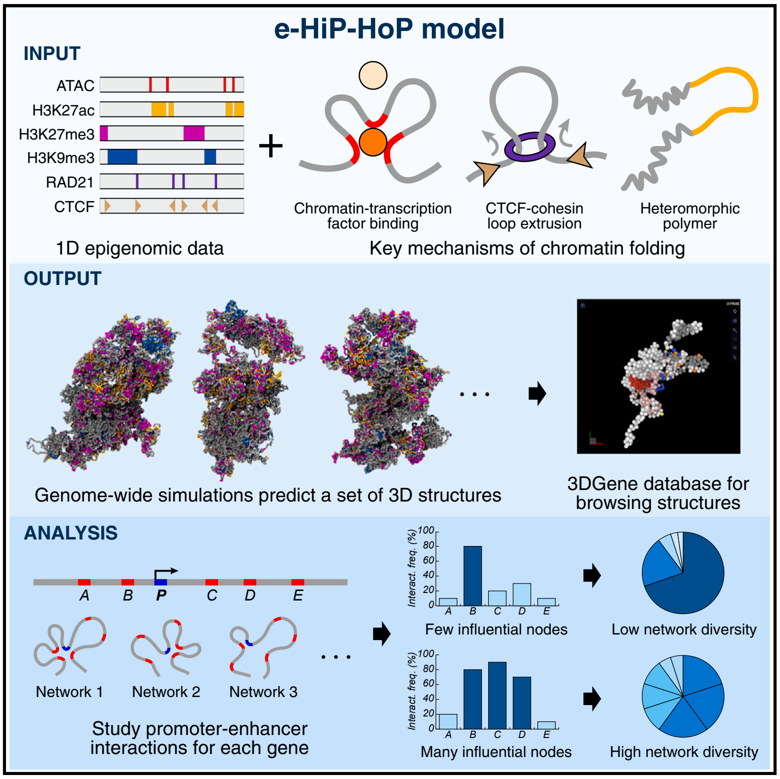

Highlights

- Polymer simulations of all active genes in GM12878 lymphoblastoid cells
- Simulations validated by high-resolution experimental datasets
- 3DGene database for searching and visualizing 3D structures
- Structural variability in chromatin is related to interaction hotspots

Authors

Michael Chiang, Chris A. Brackley, Catherine Naughton, Ryu-Suke Nozawa, Cleis Battaglia, Davide Marenduzzo, Nick Gilbert

Correspondence

davide.marenduzzo@ed.ac.uk (D.M.), nick.gilbert@ed.ac.uk (N.G.)

In brief

Chiang et al. used polymer modeling to predict the panoply of 3D structures of all active genes in human lymphoblastoid cells, depositing the information in a database: 3DGene. Mining these data, they showed that the structural diversity of genes is dependent on influential nodes—chromatin sites that frequently interact.

Chiang et al., 2024, Cell Genomics *4*, 100698
December 11, 2024 © 2024 The Authors. Published by Elsevier Inc.
https://doi.org/10.1016/j.xgen.2024.100698

CellPress

Cell Genomics

CellPress
OPEN ACCESS

Article

Genome-wide chromosome architecture prediction reveals biophysical principles underlying gene structure

Michael Chiang,[1] Chris A. Brackley,[1] Catherine Naughton,[2] Ryu-Suke Nozawa,[2,3] Cleis Battaglia,[1] Davide Marenduzzo,[1,*] and Nick Gilbert[2,4,*]

[1]SUPA, School of Physics and Astronomy, University of Edinburgh, Peter Guthrie Tait Road, Edinburgh EH9 3FD, UK

[2]MRC Human Genetics Unit, Institute of Genetics and Cancer, University of Edinburgh, Crewe Road South, Edinburgh EH4 2XU, UK

[3]Division of Experimental Pathology, Cancer Institute of the Japanese Foundation for Cancer Research (JFCR), Tokyo, Japan

[4]Lead contact

*Correspondence: davide.marenduzzo@ed.ac.uk (D.M.), nick.gilbert@ed.ac.uk (N.G.)

https://doi.org/10.1016/j.xgen.2024.100698

SUMMARY

Classical observations suggest a connection between 3D gene structure and function, but testing this hypothesis has been challenging due to technical limitations. To explore this, we developed epigenetic highly predictive heteromorphic polymer (e-HiP-HoP), a model based on genome organization principles to predict the 3D structure of human chromatin. We defined a new 3D structural unit, a "topos," which represents the regulatory landscape around gene promoters. Using GM12878 cells, we predicted the 3D structure of over 10,000 active gene topoi and stored them in the 3DGene database. Data mining revealed folding motifs and their link to Gene Ontology features. We computed a structural diversity score and identified influential nodes—chromatin sites that frequently interact with gene promoters, acting as key regulators. These nodes drive structural diversity and are tied to gene function. e-HiP-HoP provides a framework for modeling high-resolution chromatin structure and a mechanistic basis for chromatin contact networks that link 3D gene structure with function.

INTRODUCTION

In human cells, DNA is tightly wrapped around histone proteins to form a composite polymer known as chromatin. Three-dimensional (3D) chromatin folding at the level of a gene with its surrounding regulatory domain is often termed "large-scale chromatin structure." Long-standing experimental observations suggest that a link exists between such 3D folding and gene function[1]; for example, gene transcription often requires the recruitment of specific protein factors to a locus or the formation of a loop between the promoter of a gene and its enhancers.[2]

Many experimental approaches have been developed to explore chromatin folding, including fluorescence *in situ* hybridization (FISH) and Hi-C (and related techniques), but these have their limitations. First, FISH can only be used to probe the 3D positions of a limited number of gene loci, and due to the low-throughput nature of the method, it is difficult to analyze large numbers of them. High-throughput versions of FISH allow the study of thousands of loci at the same time[3,4]; however, these are still challenging to use routinely, and it is not easy to simultaneously probe expression in the same cells. Second, Hi-C can report the interaction frequency of gene loci genome wide but is limited by the spatial resolution. More importantly, this technique only provides a population average, affecting our ability to characterize the structure of individual loci; while single-cell variants exist,[5,6] the sequencing depth is, by necessity, significantly lower than that in bulk Hi-C. Recent evolutions of Hi-C, termed Micro-C and R-mC,[7] can explore chromatin structure interactions at extremely high resolution (approximately 100 bp) but still only provide a population average. Furthermore, these techniques require large amounts of cellular material. As a result of these limitations, these methods are still not ideally suitable for exploring the chromatin structure and interaction landscape of rare or difficult-to-access primary cell types.

RESULTS

e-HiP-HoP predicts 3D structures of gene topoi genome wide

Against this challenging background, we took an orthogonal approach by developing a new coarse-grained polymer model of chromatin folding. Coarse-grained models[8–11] are aimed at building simplified representations of complex systems while retaining the main biophysical interactions. Our model is evolved from the highly predictive heteromorphic polymer (HiP-HoP) framework[12] but modified to incorporate active, poised, and repressed epigenetic chromatin states, making it suitable for genome-wide studies (Figure 1A; STAR Methods). This epigenetic HiP-HoP (e-HiP-HoP) mechanistic model combines key biophysical principles for chromosome organization (Figures 1A

© 2024 The Authors. Published by Elsevier Inc.
This is an open access article under the CC BY license (http://creativecommons.org/licenses/by/4.0/).

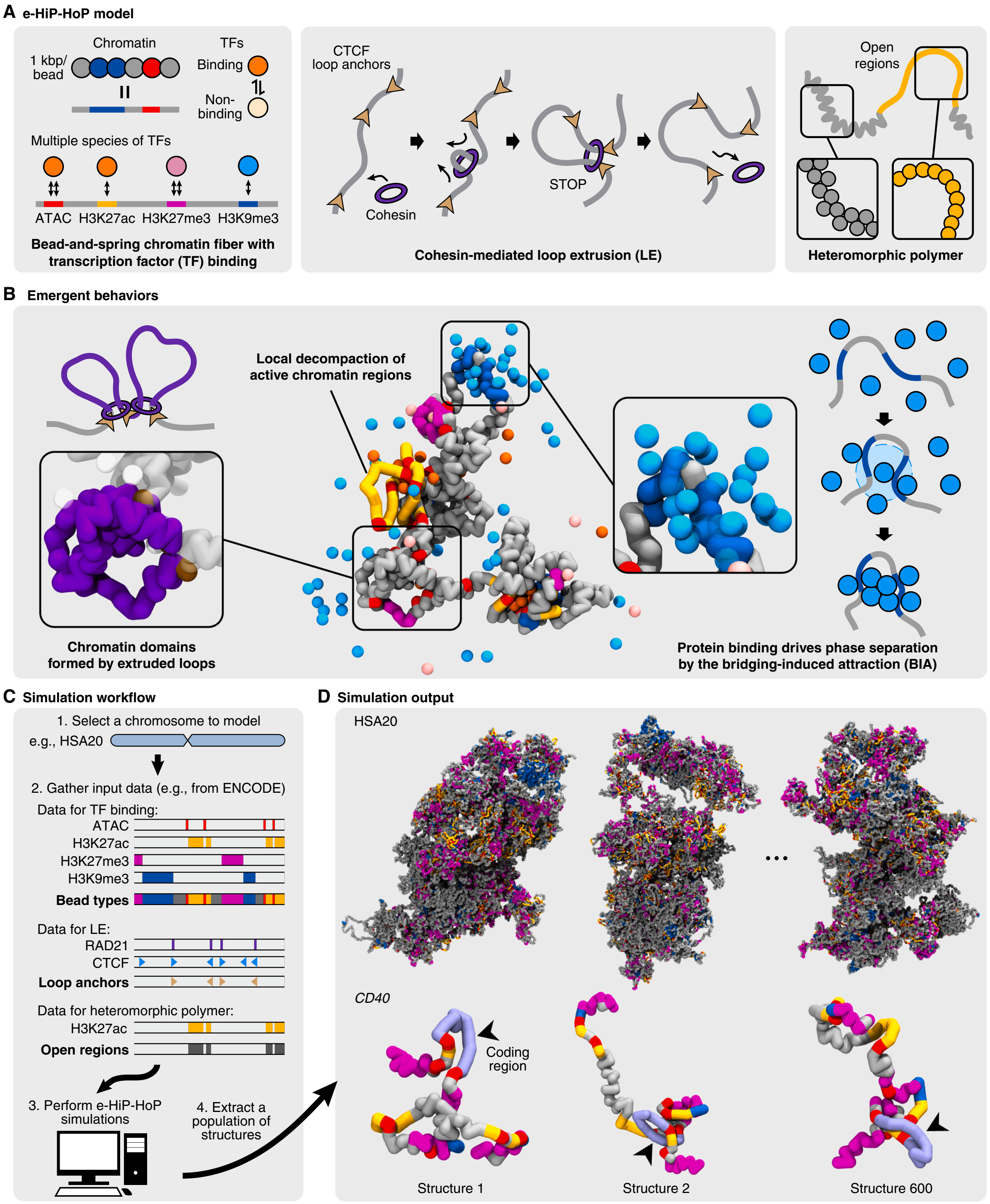

(legend on next page)

CellPress
OPEN ACCESS

and 1B). First, it includes multivalent chromatin-binding proteins, or factors, that can cluster through bridging-induced attraction[13] (Figure 1B). Specifically, these factors drive chromatin-protein-chromatin bridging, which increases local chromatin density and, in turn, facilitates more protein binding within the region, creating a positive feedback loop that leads to intranuclear microphase separation of the factors. Second, it incorporates loop extrusion by cohesin or other structural maintenance of chromosome (SMC) proteins,[14] which is necessary to explain the formation of convergent CTCF loops in mammalian genomes.[15] Third, it accounts for chromatin heteromorphism, which is required to recapitulate the decompaction of highly active genes.[12,16] Specifically, e-HiP-HoP is an extension of HiP-HoP[12] by including polycomb-like (H3K27me3) and repressive (H3K9me3) epigenetic marks, which are necessary when examining a wide spectrum of transcriptional gene states as observed in a genome-wide study.

We highlight that a key feature of e-HiP-HoP is that it does not use Hi-C or other types of 3D experimental data, unlike "inverse" modeling studies, which fit parameters to reproduce such data.[17,18] While the fits resulting from these models are excellent, they routinely require >1,000 parameters and 3D data as an input rather than a handful of parameters set by the biophysical property of chromatin and chromatin-binding proteins and 1D bioinformatic data as input. Because of this, e-HiP-HoP can be viewed as "fitting free," so that Hi-C, Micro-C, and FISH data can be used to validate the model (as they are not used as an input to the modeling). For this reason, inverse and "mechanistic" models are distinct and well suited for answering different research questions.[8,19,20]

e-HiP-HoP can, in principle, be applied to any cell types or mammalian species where there are suitable input data, although it has only thus far been applied to human and mouse chromosomes, and it is possible that the transfer to other organisms may require additional principles to be added to e-HiP-HoP. To build a baseline dataset for subsequent analysis, chromatin from GM12878 cells was modeled as 1 kbp building blocks (or "beads"), which were "painted" depending on different chromatin types[21] according to input epigenetic 1D datasets (Figures 1A and 1C; see STAR Methods for more details). Alongside this, three types of multivalent chromatin-binding factors (or transcription factors [TFs]) were considered: a generic active factor, modeling complexes of RNA polymerases and activators, and two types of repressive or silencing factors, modeling polycomb-like and heterochromatin-binding (HP1-like) proteins, respectively. The positions of binding sites for these factors along the chromatin were inferred from an assay for transposase-accessible chromatin with sequencing (ATAC-seq) dataset (for active proteins) and from chromatin immunoprecipitation sequencing (ChIP-seq) for H3K27me3 (for inactive polycomb-like proteins) and H3K9me3 (for HP1-like inactive proteins). The experimental input for loop extrusion involved using RAD21 and CTCF ChIP-seq data to identify binding sites for CTCF that act like loop anchors and halt cohesin-mediated extrusion in a binding motif orientation-specific way, while regions marked by H3K27ac were considered to form more disrupted chromatin fibers.[12]

For efficient modeling, the genome was broken into 52 fragments at gene deserts or chromosome arm boundaries (Figure S1), and polymer simulations informed by the epigenetic input data were performed on each fragment (Figure 1C; see STAR Methods for details regarding the simulation protocol and force fields). Each individual simulation iterated through 3×10^7 time steps, corresponding to approximately 40 min in real time, and at each step, the positions of all beads were recalculated. The structure of each genome fragment was simulated independently multiple times, providing a panoply of 600 structures (Figures 1D and S2A).

To abstract gene-level data from the simulated chromosome fragments, we defined a new fundamental 3D structural unit called a "topos" (plural "topoi"; STAR Methods). "Topos" derives from ancient Greek, meaning "place, region, and space," and as a mathematical concept, it is related to category theory, which deals with the study of mathematical structures and the relationships between them. In our work, we define a gene topos as the genomic region surrounding a single gene promoter that constitutes its 3D regulatory landscape; it is demarcated by the regulatory elements located farthest from the promoter that show frequent interactions with it (for a more precise definition of topos, see below). Loosely, a topos corresponds to a gene, but where a gene is a unit of heredity, a topos is a structural and functional unit. By our definition, topoi can only be defined for active genes, as inactive genes show no or very limited interactions between promoters and regulatory elements. We predicted the 3D structure of 10,742 topoi from GM12878 cells, such as *CD40* from HSA20 (Figure 1D), *BCL2* and *SERPINB8* from HSA18 (Figure S2B), and *DNMT1* and *LDLR* from HSA19 (Figure S2C).

Figure 1. Mechanistic details of the e-HiP-HoP framework for simulating 3D chromatin structures
(A) Key ingredients of the epigenetic HiP-HoP (e-HiP-HoP) framework. The chromatin fiber was modeled as a bead-and-spring polymer chain (endowed with specific properties dependent on local epigenetic marks), incorporating fundamental biophysical principles, including transcription factor (TF)-mediated bridging, loop extrusion, and variations in local chromatin fiber compaction.
(B) Simulation snapshots of the emergent behaviors observed by e-HiP-HoP: chromatin-TF interactions result in microphase separation via the bridging-induced attraction, loop extrusion leads to chromatin domains, and transcriptionally active chromatin is more flexible and disrupted.
(C) A workflow for the e-HiP-HoP framework. A chromosome fragment of interest was selected, and then the 1D epigenetic input data were collected, including ATAC-seq peaks and ChIP-seq peaks for H3K27ac, H3K27me3, and H3K9me3 (for defining TF-binding sites and locally open regions), as well as ChIP-seq peaks for RAD21 (a subunit of the cohesin complex) and CTCF (for defining loop anchors in loop extrusion). Each simulation iterated through 3×10^7 steps, and simulations were performed multiple times to determine independent structures akin to those from different cells within a population.
(D) The simulation output is an ensemble of 600 3D structures of the chromosome fragment from which individual topoi, the 3D regulatory landscapes surrounding gene promoters, were abstracted. As an example, snapshots of HSA20 (human chromosome 20) are presented with enlarged views of an example gene topos, *CD40*. Black arrowheads point to the coding region of the gene (light purple).
See also Figures S1 and S2.

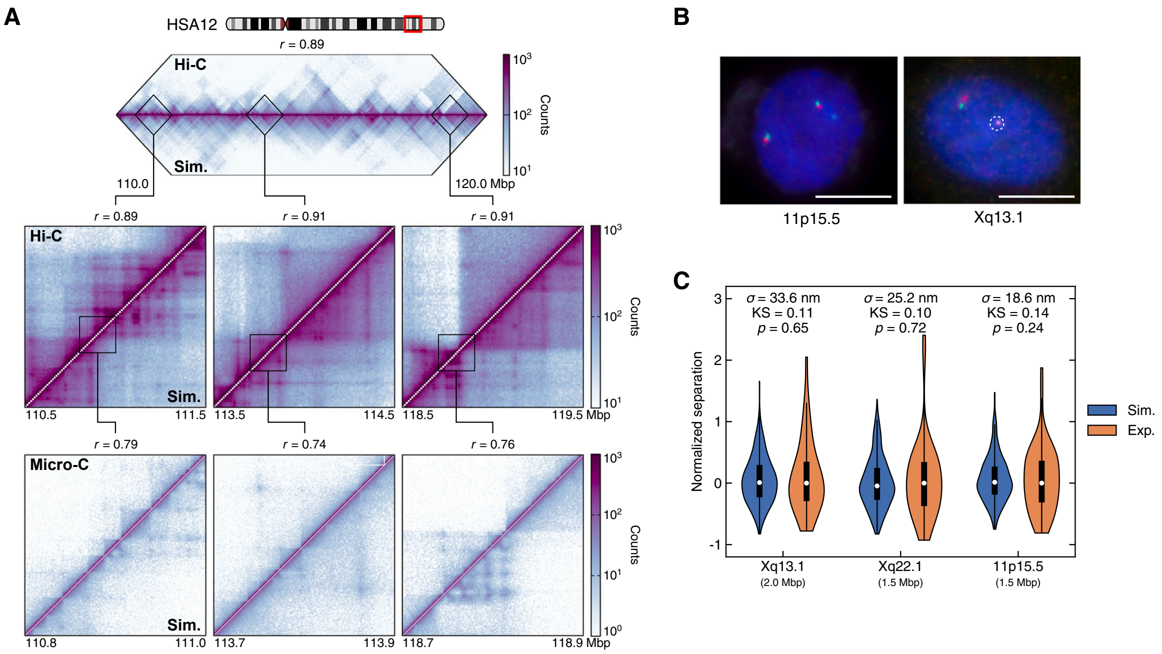

Figure 2. Chromatin structures predicted by e-HiP-HoP simulations were validated by Hi-C, Micro-C, and FISH data

(A) Comparison of contact maps between Hi-C, Micro-C, and e-HiP-HoP simulations for a 10 Mbp genomic region on chromosome HSA12. Top: comparison of the full region between Hi-C and simulations at 10 kbp resolution. Center: enlarged views of the 1 Mbp boxed regions. Bottom: comparison of the maps between Micro-C and simulations at 1 kbp resolution for the 200 kbp boxed regions marked in the center. The Pearson correlation coefficient r is reported for each comparison, and all correlations are statistically significant with $p < 10^{-10}$. More comparison examples are shown in Figure S3.

(B) Representative images of two-color FISH probes used to measure distances between selected chromosome fragments. Scale bars represent 10 μm, and the white dashed circle marks the inactive X chromosome.

(C) Violin plots comparing the shape of the normalized experimental[23] and simulated FISH distance distributions for probes in (B) and those located on Xq22.1. Here, to better reflect the local variability in chromatin compaction, we fitted the size of each chromatin bead σ locally for each probe region to experimental data to convert simulated distances into physical distances. The normalized separation is defined as the difference between the actual separation of the probes and its median, normalized by the local bead size. A two-sample Kolmogorov-Smirnov test was used to determine whether the distributions were statistically different (see STAR Methods and Figure S4 for more examples).

See also Figures S3 and S4.

After performing multiple simulations for chromosome fragments, the 3D structural data for each topos were either combined to measure average structural properties or contact statistics, analogous to averaging many cells in a population, or considered individually, akin to single-cell techniques. This unique property of high-resolution simulations enables one to analyze the panoply of 3D structures that a topos can adopt, a feat impossible using current experimental techniques.

e-HiP-HoP simulations were validated by Hi-C, Micro-C, and FISH experiments

Previously, we developed a locus-specific regulatory simulation framework termed HiP-HoP. Like e-HiP-HoP, it does not require 3D structural data as an input and, hence, can be validated by comparing its computer output predictions to existing Hi-C, Capture-C, and FISH data.[12,22] To validate e-HiP-HoP simulations in a genome-wide context, bead-bead interaction data were averaged between simulations and used to generate contact maps that were compared to Hi-C datasets from GM12878 cells[15] (Figures 2A, S3A, and S3B; STAR Methods). This comparison led to a quantitatively good agreement across genome fragments (Figures S3C–S3G), which is especially notable due to the absence of data fitting in our approach. While the agreement was statistically significant throughout the genome, some regions were predicted less well than others; for instance, patterns of contacts in gene-rich HSA19 were predicted slightly less accurately, possibly pointing either to the need to include different types of active proteins in very active regions or to the fact that transcriptionally driven chromatin decompaction is not fully captured by the model.

To probe contacts at higher resolution than Hi-C, we also compared e-HiP-HoP contact maps with Micro-C data[24] (Figures 2A, S3A, and S3B). Computing the Pearson correlation between the maps pointed to a statistically significant correlation of the patterns. Even more importantly, e-HiP-HoP correctly predicted some of the contacts between regulatory elements, missed by Hi-C due to its lower resolution. We also found even more contacts in e-HiP-HoP than in Micro-C; as the Micro-C data are still quite sparse in view of the much enhanced experimental resolution, it would be of interest to perform targeted experiments to determine whether these additional predicted contacts can form *in vivo*. The combined comparison with Hi-C and Micro-C validates the predictions of e-HiP-HoP for cell populations genome wide and indicates that it can recapitulate the

formation of interactions between regulatory elements by predicting chromatin-chromatin contacts at high resolution.

To validate e-HiP-HoP predictions with single-cell rather than population-averaged experimental data, distances between pairs of chromatin regions in the simulations were used to generate probability distributions, which were compared to those from experimentally generated two-color FISH data (Figure 2B). Regions of the genome with diverse characteristics, such as gene density and transcriptional activity, were selected and compared, with the distributions being well matched (Figures 2C and S4). Despite the large number of parameters that are thought to be important for determining large-scale chromatin structure in nuclei, e-HiP-HoP can account fairly well for transcriptionally dependent chromatin decompaction and, in general, recapitulate 3D distances within gene-regulatory domains. In addition to the mean distances being well matched, the shapes of the distributions are also consistent. However, to account for all datasets, it was necessary to fit the size of a chromatin bead locally for each probe region, which suggests that the effective thickness of chromatin at the kbp scale changes slightly with chromatin context. Possibly, this points to additional mechanisms of transcriptionally dependent chromatin folding not included in e-HiP-HoP, such as DNA supercoiling[25] or interactions with other nuclear structures (e.g., nuclear bodies[26] or the nuclear mesh[27]). Additionally, in most cases, the experimental data show a broader distribution with a small number of outliers. These issues could be due to experimental artifacts (e.g., nuclear distortion in the experiments) or, alternatively, due to a small number of loci within a population of cells showing an extended organization.

The 3DGene database provides a platform for visualizing 3D structures

e-HiP-HoP simulates large chromosome fragments (Figure S1). As *cis*-regulatory element interactions, such as those between promoters and enhancers, are thought to closely link to function,[2] the regulatory domain surrounding individual promoters of each active gene, corresponding to its associated topos, was predicted from simulations. The simulated 3D landscapes surrounding genes from e-HiP-HoP were deposited within a newly developed, publicly available database named 3DGene (https://3dgene.igc.ed.ac.uk; Figure 3), which provides a platform to enable researchers to interactively view 3D topos structures and Hi-C-like contact maps for any active human gene. Genes can be searched by using different identifiers, such as Ensembl ID or gene name, and simulation data for different genomic regions can be downloaded as required.

The 3DGene database contains structures of 10,742 active gene topoi in the GM12878 cell line, and the most frequently observed structures of each topos are shown in the interactive viewer window (Figure 3D). In contrast to studies focusing on specific gene loci, the large number of topoi included in the database offers the statistical power to discover generic links between structure and function by comparing the predicted structures with other bioinformatic datasets; for instance, on Gene Ontology. The database is currently populated with topoi structures from GM12878, but it has the capacity to take data from other cell lines, tissues, or different species as they become available.

Influential nodes are linked to gene function

To systematically parameterize the 3D structures in the vicinity of genes, we identified the chromatin beads mapping to gene promoters[28] and then examined the 3D interactions between each promoter and beads covering ATAC peaks (henceforth referred to as "ATAC beads"; Figures 3E and 4A). ATAC peaks were chosen as they identify open chromatin and strongly correlate with regulatory elements, such as enhancers. For example, in Figure 4A, promoter *P* often interacts with the regulatory elements *A*, *B*, and *C*. ATAC beads (such as *A*, *B*, and *C*) that were proximate to a promoter in $\geq$10% of the simulated structures were designated as "ATAC partners" (Figure 4A). A topos is then defined in our work as the genomic region encompassing all ATAC partners of a gene promoter. The median size of topoi was 209 kbp, significantly smaller than the average $\sim$1 Mbp size of topologically associating domains (TADs), indicating that the two are distinct structural units. Interestingly, this size is similar to the median CTCF-cohesin loop size,[15] although not all topoi contained ATAC sites corresponding to CTCF binding sites.

In order to identify structural features that are functionally relevant, a useful concept is that of "influential nodes." These were defined as ATAC partners that are in contact with a gene promoter very frequently ($\geq$50% of all simulated structures; Figure 4A) and, hence, expected to be key regulators of expression. Gene topoi across the genome contain a highly variable number of partners and influential nodes (Figures 4B and 4C). Comparing a sample of genes with 14 partners, the two extremes correspond to *ZBTB5*, a housekeeping gene coding for a zinc-finger protein without any influential nodes, and *PTPN22*, a lymphoid-specific gene coding for an intracellular phosphatase with seven influential nodes (Figures 4C and 4D). Genome wide, the distribution of the number of influential nodes is a monotonically decreasing function. While 1D proximity to the promoter is an important factor, the identification of an ATAC site as an influential node also strongly depends on local chromatin context.

To test whether influential nodes correspond to function, we compared Gene Ontology (GO) biological function terms between genes with different numbers of influential nodes in their topos (Figures 4E and 4F). The results showed that genes with more influential nodes are overrepresented with GO terms that are linked to tissue-specific functions (i.e., immune response), while those with few or none are more likely to be housekeeping or tissue invariant. This indicates that the number of influential nodes is a structural feature with immediate relevance to transcription and cell function.

Topos structural variability correlates with the number of influential nodes

To better characterize the structure of a gene topos, we defined a local chromatin interaction network (hereafter simply referred to as a "network") as a unique subset of ATAC partners that interact with the promoter for a given structure (Figure 5A) and cataloged the observed networks of all gene topoi. The number of networks of a gene topos correlates strongly with its number of partners, while there was large heterogeneity between topoi with similar numbers of partners (Figure 5B). To quantify the structural diversity of a given topos, we then used a concept

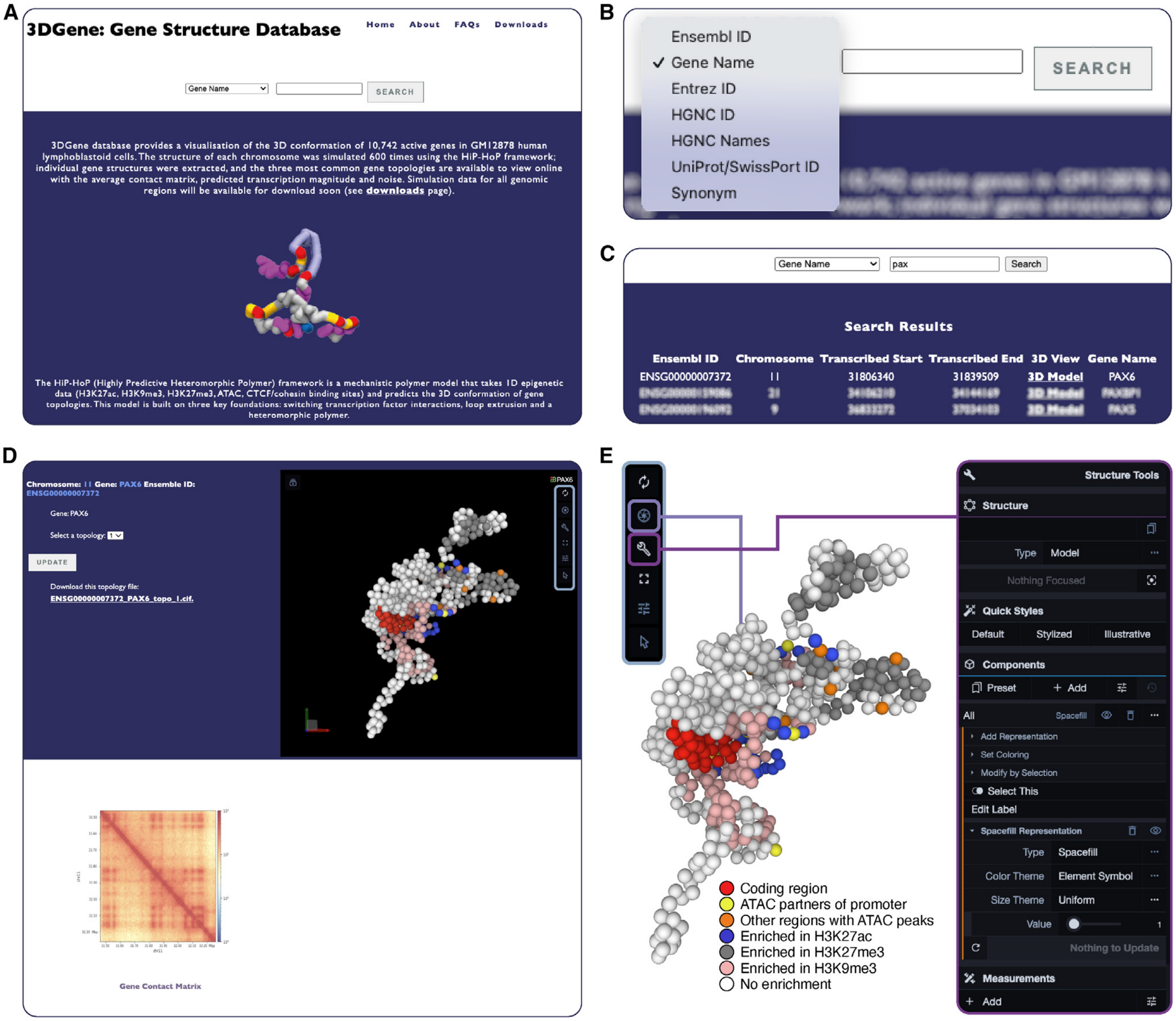

Figure 3. 3DGene website provides a simple interface for browsing and downloading simulated structures predicted by e-HiP-HoP

(A) Interface to the 3DGene database at https://3dgene.igc.ed.ac.uk. The database enables visualization of 10,742 active gene topoi in GM12878 human lymphoblastoid cells.

(B) Genes can be searched using various identifiers, including their Ensembl ID and common name.

(C) After searching for a gene using either a full or partial identifier, hits are returned with chromosome and gene coordinates (hg19). Selecting the "3D Model" link reveals an interactive results page.

(D) The results page has three main elements: gene details, interactive structure viewing window, and contact matrix. From the gene details panel, one of the three most common 3D structures of the gene topos can be selected and examined in the viewing panel, and a hyperlink is provided to download the coordinates of the structure as a crystallographic information file (CIF). Each bead corresponds to 1 kbp of chromatin, and bead colors are explained in the enlarged snapshot on the right.

(E) The 3D structure can be rotated and magnified or reduced using the mouse. To the right of the viewing window is the control panel (highlighted in light blue). Here, the aperture icon is used for capturing a screen grab, while the spanner reveals further visualization settings. Below the gene details panel is the contact matrix within the gene topos, constructed from all simulated structures.

borrowed from information theory and defined diversity (*H*) as the Shannon entropy normalized by the number of partners in the topos, which measures the relative distribution of individual networks (Figures 5A and 5C).

Our analysis indicated that, across the genome, there was large variability in the network diversity between gene topoi (Figure 5C). For example, the topos of *SMARCA5*, encoding a component of the chromatin remodeling and spacing factor (RSF) facilitating RNA polymerase II transcription, has eight ATAC partners and 56 different networks. The most common network accounts for over 60% of all simulated structures, and concomitantly, *SMARCA5* has low structural diversity

CellPress
OPEN ACCESS

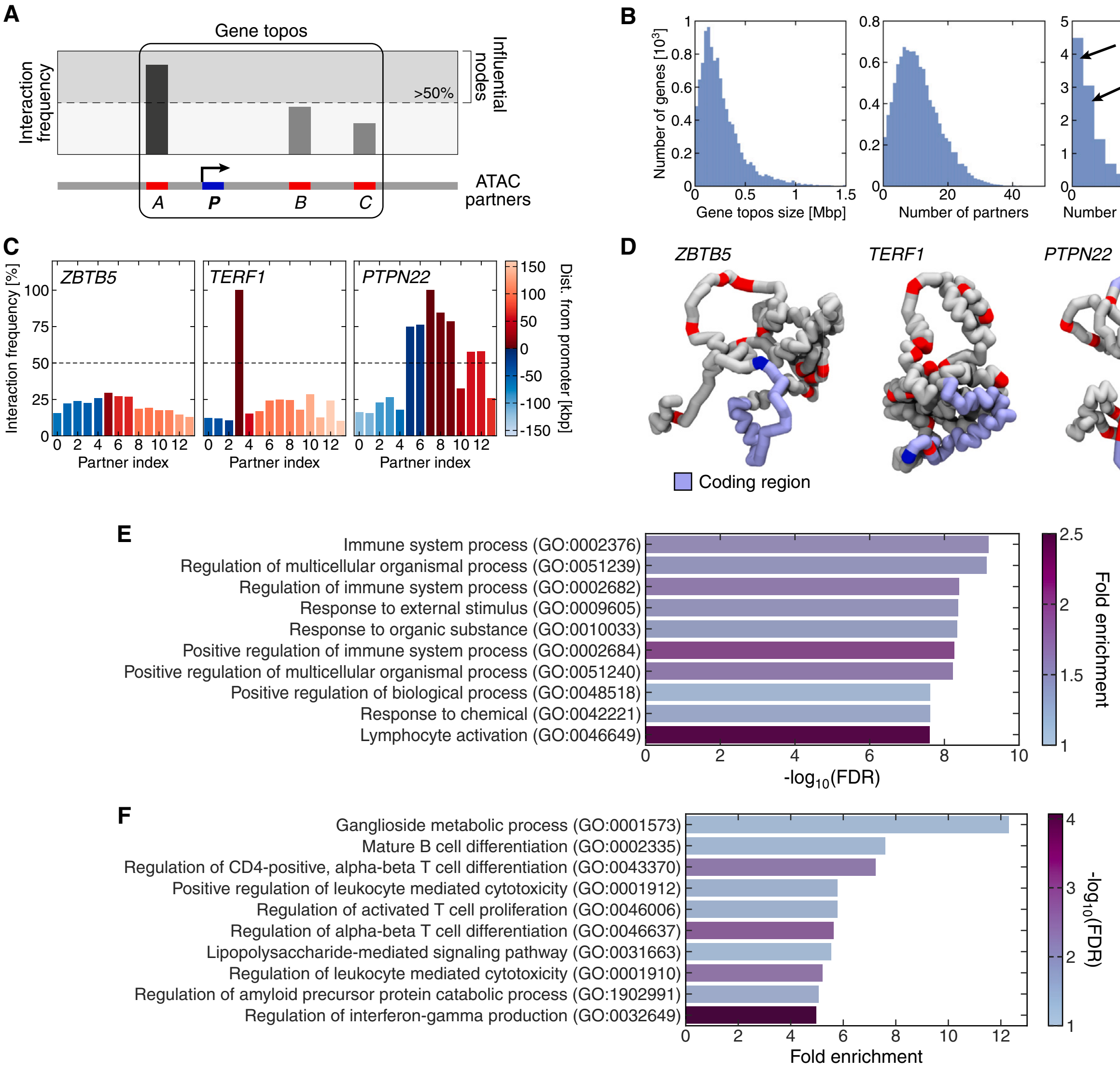

Figure 4. Influential nodes are linked to gene function

(A) A schematic illustrates how ATAC partners and influential nodes are determined for each gene promoter. In our notation, a topos is defined as the contiguous chromatin region encompassing all partners of its promoter, which essentially corresponds to the regulatory landscape. The box highlights the topos corresponding to the gene whose promoter is *P*.

(B) Histograms showing the distributions of size, number of partners, and number of influential nodes for gene topoi genome wide.

(C) The interaction frequency of each ATAC partner in the topoi of three genes with 14 partners: *ZBTB5*, *TERF1*, and *PTPN22*. These topoi have zero, one, and seven influential nodes, respectively (the dashed line marks the threshold for a partner to be considered as an influential node). The color scale indicates the distance between each partner and the promoter.

(D) Examples of 3D structure for the topoi described in (C).

(E and F) A list of the top ten Gene Ontology (GO) biological function terms, ranked by (E) $-\log_{10}$ of the false discovery rate (FDR) or (F) fold enrichment, from performing an overrepresentation test in GO terms comparing genes with more than one influential node to those with only a single node. Most of these GO terms are related to immune response; since we simulated lymphoblastoid cells, this result indicates that genes with a higher number of influential nodes are typically more tissue specific. The test was completed using the web tool Protein Analysis Through Evolutionary Relationship (PANTHER) and the GO database released on November 16, 2021.

(Figure 5D). In contrast, *GINS4*, encoding SLD5, a key component of the DNA replication complex, has the same number of partners but 89 networks, with the most common one accounting for just 14.7% of the simulated structures, corresponding to high diversity (Figure 5E).

To understand what mechanisms might determine the degree of network diversity of a gene topos, we systematically correlated the *H* score with other structural features found computationally. This analysis showed that a large proportion of influential partners (or nodes) correlated significantly with diversity

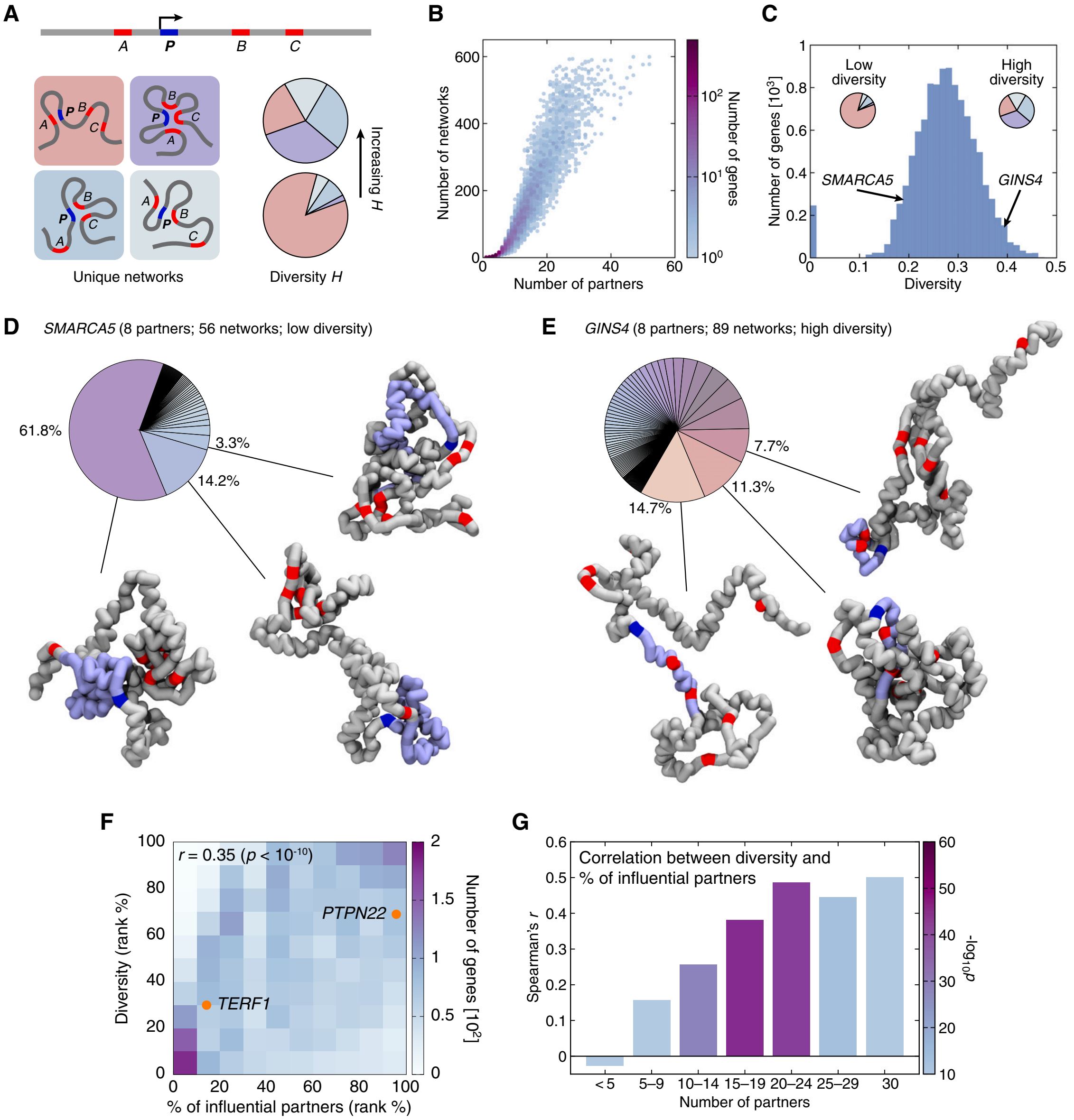

Figure 5. Intra-topos interactions and influential nodes determine structural diversity

(A) An illustration explaining the identification of networks in a gene topos and the associated diversity *H* score. Here, with a population of four networks, a higher *H* is achieved when the sampled structures are distributed more evenly among the networks (i.e., more equal slices in the pie chart), whereas a lower *H* occurs when many structures are associated with one of the networks (i.e., more unequal slices).

(B) A scatterplot showing the number of networks against the number of partners for topoi genome wide.

(C) A histogram showing the distribution of structural diversity for topoi genome wide.

(D) Network pie chart and example structures for the three most frequent networks for *SMARCA5*, a low-diversity topos with eight partners and 56 networks.

(E) Similar to (D) but for *GINS4*, a high-diversity topos with eight partners and 89 networks.

(F) A 2D histogram showing the correlation between structural diversity and the percentage of ATAC partners being influential for genes with at least one influential node, and not all partners are influential (Spearman correlation coefficient *r* is reported). Circles indicate the positions of two example genes, *TERF1* and *PTPN22*, with the former having a smaller proportion of influential partners (Figure 4C).

(G) A plot showing the same correlation between diversity and the percentage of influential partners, binned according to the total number of partners of the genes. The color of each bar indicates the *p* value of the correlation.

(Figure 5F). This correlation was most statistically significant in gene topoi with an intermediate (10–24) number of partners (Figure 5G) and arises because having a large number of influential nodes introduces more wiring options for choosing edges in the interaction network, which, in turn, yields a larger number of available chromatin looping configurations. Interestingly, the number of influential nodes correlated with both GO and structural diversity, and we speculate that the larger degree of

CellPress
OPEN ACCESS

structural heterogeneity may be beneficial for the robustness of a tissue-specific gene, as stochastic inactivation of one influential node is unlikely to silence its activity.

DISCUSSION

We developed e-HiP-HoP, a simulation framework to predict 3D chromatin structure genome wide on the basis of a limited set of epigenetic marks and the location of accessible chromatin regions (mapped by ATAC-seq). e-HiP-HoP was validated by comparing its structural predictions to experimental Hi-C, Micro-C, and FISH data and then used to simulate the 3D chromatin structure of all genes in human lymphoblastoid cells. Although this study used GM12878 lymphoblastoid cells, the e-HiP-HoP framework can be used to generate chromatin structure profiles for genes in difficult-to-study or rare cell types, with the only requirement being the available input epigenetic marks (Figure 1C). This framework is also suitable for analyzing gene structure in other mammalian species, such as the mouse,[12] without modification, and we anticipate that, with minor adaption, it could also be used to analyze gene structures in other higher eukaryotes, such as *Drosophila*, *C. elegans*, or zebrafish. In this study, we focused our analysis on shorter-range interactions at the scale of individual genes. However, as the e-HiP-HoP simulations were undertaken on large chromosomal fragments (Figure S1A), long-range *cis* interactions were also captured; yet, these are at a very low frequency compared to short-range interactions. Additionally, this framework cannot capture *trans* interactions that occur between chromosomes or across chromosome arms that were simulated separately.

All simulation results were stored in a new database called 3DGene, a resource that is available to the wider research community. By mining 3DGene structures and comparing to other datasets on gene identity and function, the functional roles of any 3D structural features found computationally can be inferred. At present, 3DGene has the functionality to visualize the top three most commonly observed interaction networks for a gene, but we anticipate that, as the resource is populated with more tools, additional analysis modules will be added to enable more comprehensive comparisons between gene structures. For more complex offline bespoke analysis, 3DGene also allows researchers to download all simulated trajectories for individual genes, genomic regions, or chromosome fragments.

Our results indicate that the network of chromatin interactions (or contacts) between regulatory elements that arise through the bridging-induced attraction[13] and loop extrusion[14] forms a new fundamental 3D structural unit, which we term a topos (median size: 209 kbp). Although the boundaries of the topoi were determined by mining interaction data for each gene region, by analogy, similar information could be extracted from high-resolution Hi-C data (Figure 2A) or by analyzing Capture-C data, as we have done previously for the *Pax6* locus.[12] From this definition of a structural regulatory unit, we developed the concept of influential nodes, which are the regulatory elements in a gene topos with very frequent interactions with the promoter of that gene (Figures 4 and 5). Influential nodes play key roles in many aspects of network theory. For instance, identifying influential nodes is key to understanding the dynamics of epidemics or virus spreading in social networks or the internet[29] or to discover drug target candidates in a protein or DNA-protein interactome network.[30] In our case, the number of influential nodes correlates significantly with GO so that gene topoi with a higher number of influential nodes are more often tissue specific. These results are consistent with immuno genome architecture mapping (ImmunoGAM) experiments,[31] which suggest that chromatin networks are cell type specific, at variance with TADs, which are largely cell type insensitive.[32]

The number of influential nodes also determines the structural diversity of a gene topos, which measures the variability in the looping networks associated with interactions between promoters and regulatory elements. What mechanisms underlie this correlation? Exploration of the data ascribes this to the larger possible number of rewiring events between regulatory elements, which arises as the number of influential nodes increases. We speculate that, for tissue-specific genes, having a large number of influential nodes and a higher structural variability may be evolutionarily advantageous. First, having many influential nodes may render these genes more fault tolerant. For instance, losing one influential node in one cell (e.g., due to binding of a silencing protein) may not completely inactivate its associated gene because of the possibility of rescue from other available nodes in the topos. Second, if structures and expression are linked, an increased structural variability leads to an additional avenue to tune the transcriptional activity of tissue-specific genes with respect to tissue-invariant ones, which may be beneficial for genes whose expression should be modulated through development. A goal for future research is to experimentally validate structural heterogeneity by single-cell chromatin-tracing techniques, such as optical reconstruction of chromatin architecture (ORCA),[33,34] and by single-cell Hi-C.[6] We also plan to further test these ideas; for instance, by measuring the variability in expression or transcriptional activity of genes with different predicted degrees of structural variability. Additionally, alterations in epigenetic programs that occur through genetic mutations could impact the influential nodes and structural variabilities of only certain types of genes in cells and tissues,[35,36] thereby providing a potential molecular basis for disease mechanisms.

Limitations of the study

While e-HiP-HoP simulations have enabled us to explore the 3D structure of all active genes across human lymphoblastoid cells, there are limitations on the structure predictions with the current modeling framework, which we aim to address in future work. First, owing to computational cost, we simulated the chromosomes in segments, and so the model cannot account for the effect of *trans* interactions and ultra-long-range *cis* interactions, which could play a role in regulating some genes. This could be resolved in the future as more powerful computers and more efficient algorithms become available so that it is less expensive to simulate longer segments and, indeed, multiple chromosomes simultaneously. Second, when validating the model by comparing simulated and experimental FISH data, we observed that the two datasets are more closely matched when we allow the chromatin bead size in our simulations to vary between different loci, suggesting that the model has yet

to fully capture the variability in local compaction of chromatin. This could be improved by introducing more levels of compaction or disruption to the simulated chromatin fiber rather than the two levels implemented currently. Finally, there are other mechanisms influencing gene structure, such as those driven by transcription, that we have yet to investigate with e-HiP-HoP. For instance, nuclear RNAs can interact with scaffolding proteins, such as SAF-A, and alter the local environment around actively transcribed chromatin and its conformation.[27,37,38] Additionally, transcriptionally induced supercoiling can also lead to large-scale changes in chromatin folding.[39] All of these are exciting avenues for further development of e-HiP-HoP.

RESOURCE AVAILABILITY

Lead contact

Requests for further information, resources, and reagents should be directed to and will be fulfilled by the lead contact, Nick Gilbert (nick.gilbert@ed.ac.uk).

Materials availability

This study did not generate new unique reagents.

Data and code availability

The datasets generated in the current study and the Large-scale Atomic/Molecular Massively Parallel Simulator (LAMMPS) scripts for running e-HiP-HoP are on Edinburgh DataShare (https://doi.org/10.7488/ds/7821).

ACKNOWLEDGMENTS

We thank Ewan McDowall for developing the 3DGene database, Craig Nicol for designing the web page, and the Edinburgh Compute and Data Facility (ECDF; http://www.ecdf.ed.ac.uk/). Thanks also to our group members and colleagues, in particular Javier Caceres and Martin Taylor, who provided advice during the project, and Jim Allan and James Ding for comments on the manuscript. This work was funded by the European Research Council (ERC CoG 648050 THREEDCELLPHYSICS), UK Medical Research Council (MR/J00913X/1 and MC_UU_00007/13), and the Wellcome Trust (223097/Z/21/Z).

AUTHOR CONTRIBUTIONS

M.C., C.A.B., D.M., and N.G. designed research. M.C., C.A.B., and C.B. performed simulations. C.N. and R.-S.N. performed lab-based research. M.C., C.A.B., D.M., and N.G. wrote the manuscript with input from all authors.

DECLARATION OF INTERESTS

The authors declare no competing interests.

STAR★METHODS

Detailed methods are provided in the online version of this paper and include the following:

- KEY RESOURCES TABLE
- METHOD DETAILS
  - Polymer modeling
  - The chromatin fiber
  - Multivalent chromatin-binding proteins
  - Loop extrusion
  - Heteromorphic chromatin modeling
  - Equations of motion
  - Simulation units and parameters
  - Initialization and simulation protocol
  - Simulation input data
  - Comparison with Hi-C, Micro-C, and FISH experiments
  - Comparison of contact maps between simulations, Hi-C, and Micro-C
  - Comparison with FISH experiments
  - Analysis of 3D simulated chromatin structure
  - Shannon entropy and structural diversity
- QUANTIFICATION AND STATISTICAL ANALYSIS

SUPPLEMENTAL INFORMATION

Supplemental information can be found online at https://doi.org/10.1016/j.xgen.2024.100698.

Received: November 14, 2023
Revised: September 4, 2024
Accepted: October 28, 2024
Published: November 25, 2024

REFERENCES

1. Kempfer, R., and Pombo, A. (2020). Methods for mapping 3D chromosome architecture. Nat. Rev. Genet. *21*, 207–226. https://doi.org/10.1038/s41576-019-0195-2.
2. Schoenfelder, S., and Fraser, P. (2019). Long-range enhancer-promoter contacts in gene expression control. Nat. Rev. Genet. *20*, 437–455. https://doi.org/10.1038/s41576-019-0128-0.
3. Su, J.-H., Zheng, P., Kinrot, S.S., Bintu, B., and Zhuang, X. (2020). Genome-Scale Imaging of the 3D Organization and Transcriptional Activity of Chromatin. Cell *182*, 1641–1659.e26. https://doi.org/10.1016/j.cell.2020.07.032.
4. Takei, Y., Yun, J., Zheng, S., Ollikainen, N., Pierson, N., White, J., Shah, S., Thomassie, J., Suo, S., Eng, C.-H.L., et al. (2021). Integrated spatial genomics reveals global architecture of single nuclei. Nature *590*, 344–350. https://doi.org/10.1038/s41586-020-03126-2.
5. Rappoport, N., Chomsky, E., Nagano, T., Seibert, C., Lubling, Y., Baran, Y., Lifshitz, A., Leung, W., Mukamel, Z., Shamir, R., et al. (2023). Single cell Hi-C identifies plastic chromosome conformations underlying the gastrulation enhancer landscape. Nat. Commun. *14*, 3844. https://doi.org/10.1038/s41467-023-39549-4.
6. Nagano, T., Lubling, Y., Várnai, C., Dudley, C., Leung, W., Baran, Y., Mendelson Cohen, N., Wingett, S., Fraser, P., and Tanay, A. (2017). Cell-cycle dynamics of chromosomal organization at single-cell resolution. Nature *547*, 61–67. https://doi.org/10.1038/nature23001.
7. Goel, V.Y., Huseyin, M.K., and Hansen, A.S. (2023). Region Capture Micro-C reveals coalescence of enhancers and promoters into nested microcompartments. Nat. Genet. *55*, 1048–1056. https://doi.org/10.1038/s41588-023-01391-1.
8. Brackey, C.A., Marenduzzo, D., and Gilbert, N. (2020). Mechanistic modeling of chromatin folding to understand function. Nat. Methods *17*, 767–775. https://doi.org/10.1038/s41592-020-0852-6.
9. Barbieri, M., Chotalia, M., Fraser, J., Lavitas, L.-M., Dostie, J., Pombo, A., and Nicodemi, M. (2012). Complexity of chromatin folding is captured by the strings and binders switch model. Proc. Natl. Acad. Sci. USA *109*, 16173–16178. https://doi.org/10.1073/pnas.1204799109.
10. Jost, D., Carrivain, P., Cavalli, G., and Vaillant, C. (2014). Modeling epigenome folding: formation and dynamics of topologically associated chromatin domains. Nucleic Acids Res. *42*, 9553–9561. https://doi.org/10.1093/nar/gku698.
11. Chiariello, A.M., Annunziatella, C., Bianco, S., Esposito, A., and Nicodemi, M. (2016). Polymer physics of chromosome large-scale 3D organisation. Sci. Rep. *6*, 29775. https://doi.org/10.1038/srep29775.
12. Buckle, A., Brackley, C.A., Boyle, S., Marenduzzo, D., and Gilbert, N. (2018). Polymer Simulations of Heteromorphic Chromatin Predict the 3D

CellPress
OPEN ACCESS

Folding of Complex Genomic Loci. Mol. Cell *72*, 786–797.e11. https://doi.org/10.1016/j.molcel.2018.09.016.

13. Brackley, C.A., Taylor, S., Papantonis, A., Cook, P.R., and Marenduzzo, D. (2013). Nonspecific bridging-induced attraction drives clustering of DNA-binding proteins and genome organization. Proc. Natl. Acad. Sci. USA *110*, E3605–E3611. https://doi.org/10.1073/pnas.1302950110.
14. Fudenberg, G., Imakaev, M., Lu, C., Goloborodko, A., Abdennur, N., and Mirny, L.A. (2016). Formation of Chromosomal Domains by Loop Extrusion. Cell Rep. *15*, 2038–2049. https://doi.org/10.1016/j.celrep.2016.04.085.
15. Rao, S.S.P., Huntley, M.H., Durand, N.C., Stamenova, E.K., Bochkov, I.D., Robinson, J.T., Sanborn, A.L., Machol, I., Omer, A.D., Lander, E.S., and Aiden, E.L. (2014). A 3D Map of the Human Genome at Kilobase Resolution Reveals Principles of Chromatin Looping. Cell *159*, 1665–1680. https://doi.org/10.1016/j.cell.2014.11.021.
16. Benabdallah, N.S., Williamson, I., Illingworth, R.S., Kane, L., Boyle, S., Sengupta, D., Grimes, G.R., Therizols, P., and Bickmore, W.A. (2019). Decreased Enhancer-Promoter Proximity Accompanying Enhancer Activation. Mol. Cell. *76*, 473–484.e7. https://doi.org/10.1016/j.molcel.2019.07.038.
17. Qi, Y., and Zhang, B. (2019). Predicting three-dimensional genome organization with chromatin states. PLoS Comput. Biol. *15*, e1007024. https://doi.org/10.1371/journal.pcbi.1007024.
18. Bianco, S., Lupiáñez, D.G., Chiariello, A.M., Annunziatella, C., Kraft, K., Schöpflin, R., Wittler, L., Andrey, G., Vingron, M., Pombo, A., et al. (2018). Polymer physics predicts the effects of structural variants on chromatin architecture. Nat. Genet. *50*, 662–667. https://doi.org/10.1038/s41588-018-0098-8.
19. Chiang, M., Brackley, C.A., Marenduzzo, D., and Gilbert, N. (2022). Predicting genome organisation and function with mechanistic modelling. Trends Genet. *38*, 364–378. https://doi.org/10.1016/j.tig.2021.11.001.
20. Chiang, M., Forte, G., Gilbert, N., Marenduzzo, D., and Brackley, C.A. (2022). Predictive Polymer Models for 3D Chromosome Organization. Methods Mol. Biol. *2301*, 267–291. https://doi.org/10.1007/978-1-0716-1390-0_14.
21. Filion, G.J., van Bemmel, J.G., Braunschweig, U., Talhout, W., Kind, J., Ward, L.D., Brugman, W., de Castro, I.J., Kerkhoven, R.M., Bussemaker, H.J., and van Steensel, B. (2010). Systematic Protein Location Mapping Reveals Five Principal Chromatin Types in Drosophila Cells. Cell *143*, 212–224. https://doi.org/10.1016/j.cell.2010.09.009.
22. Brackley, C.A., Brown, J.M., Waithe, D., Babbs, C., Davies, J., Hughes, J.R., Buckle, V.J., and Marenduzzo, D. (2016). Predicting the three-dimensional folding of cis-regulatory regions in mammalian genomes using bioinformatic data and polymer models. Genome Biol. *17*, 59. https://doi.org/10.1186/s13059-016-0909-0.
23. Naughton, C., Sproul, D., Hamilton, C., and Gilbert, N. (2010). Analysis of Active and Inactive X Chromosome Architecture Reveals the Independent Organization of 30 nm and Large-Scale Chromatin Structures. Mol. Cell *40*, 397–409. https://doi.org/10.1016/j.molcel.2010.10.013.
24. Krietenstein, N., Abraham, S., Venev, S.V., Abdennur, N., Gibcus, J., Hsieh, T.-H.S., Parsi, K.M., Yang, L., Maehr, R., Mirny, L.A., et al. (2020). Ultrastructural Details of Mammalian Chromosome Architecture. Mol. Cell *78*, 554–565.e7. https://doi.org/10.1016/j.molcel.2020.03.003.
25. Gilbert, N., and Allan, J. (2014). Supercoiling in DNA and chromatin. Curr. Opin. Genet. Dev. *25*, 15–21. https://doi.org/10.1016/j.gde.2013.10.013.
26. Mao, Y.S., Zhang, B., and Spector, D.L. (2011). Biogenesis and function of nuclear bodies. Trends Genet. *27*, 295–306. https://doi.org/10.1016/j.tig.2011.05.006.
27. Nozawa, R.-S., Boteva, L., Soares, D.C., Naughton, C., Dun, A.R., Buckle, A., Ramsahoye, B., Bruton, P.C., Saleeb, R.S., Arnedo, M., et al. (2017). SAF-A Regulates Interphase Chromosome Structure through Oligomerization with Chromatin-Associated RNAs. Cell *169*, 1214–1227.e18. https://doi.org/10.1016/j.cell.2017.05.029.
28. Dreos, R., Ambrosini, G., Groux, R., Cavin Périer, R., and Bucher, P. (2017). The eukaryotic promoter database in its 30th year: focus on non-vertebrate organisms. Nucleic Acids Res. *45*, D51–D55. https://doi.org/10.1093/nar/gkw1069.
29. Cohen, R., Havlin, S., and ben-Avraham, D. (2003). Efficient Immunization Strategies for Computer Networks and Populations. Phys. Rev. Lett. *91*, 247901. https://doi.org/10.1103/physrevlett.91.247901.
30. Sathyapriya, R., Vijayabaskar, M.S., and Vishveshwara, S. (2008). Insights into Protein-DNA Interactions through Structure Network Analysis. PLoS Comput. Biol. *4*, e1000170. https://doi.org/10.1371/journal.pcbi.1000170.
31. Winick-Ng, W., Kukalev, A., Harabula, I., Zea-Redondo, L., Szabó, D., Meijer, M., Serebreni, L., Zhang, Y., Bianco, S., Chiariello, A.M., et al. (2021). Cell-type specialization is encoded by specific chromatin topologies. Nature *599*, 684–691. https://doi.org/10.1038/s41586-021-04081-2.
32. Dixon, J.R., Selvaraj, S., Yue, F., Kim, A., Li, Y., Shen, Y., Hu, M., Liu, J.S., and Ren, B. (2012). Topological domains in mammalian genomes identified by analysis of chromatin interactions. Nature *485*, 376–380. https://doi.org/10.1038/nature11082.
33. Mateo, L.J., Sinnott-Armstrong, N., and Boettiger, A.N. (2021). Tracing DNA paths and RNA profiles in cultured cells and tissues with ORCA. Nat. Protoc. *16*, 1647–1713. https://doi.org/10.1038/s41596-020-00478-x.
34. Hafner, A., and Boettiger, A. (2023). The spatial organization of transcriptional control. Nat. Rev. Genet. *24*, 53–68. https://doi.org/10.1038/s41576-022-00526-0.
35. Khazaei, S., de Jay, N., Deshmukh, S., Hendrikse, L.D., Jawhar, W., Chen, C.C.L., Mikael, L.G., Faury, D., Marchione, D.M., Lanoix, J., et al. (2020). H3.3 G34W Promotes Growth and Impedes Differentiation of Osteoblast-Like Mesenchymal Progenitors in Giant Cell Tumor of Bone. Cancer Discov. *10*, 1968–1987. https://doi.org/10.1158/2159-8290.CD-20-0461.
36. Krug, B., de Jay, N., Harutyunyan, A.S., Deshmukh, S., Marchione, D.M., Guilhamon, P., Bertrand, K.C., Mikael, L.G., McConechy, M.K., Chen, C.C.L., et al. (2019). Pervasive H3K27 Acetylation Leads to ERV Expression and a Therapeutic Vulnerability in H3K27M Gliomas. Cancer Cell *35*, 782–797.e8. https://doi.org/10.1016/j.ccell.2019.04.004.
37. Marenda, M., Michieletto, D., Czapiewski, R., Stocks, J., Winterbourne, S.M., Miles, J., Fleming, O.C.A., Lazarova, E., Chiang, M., Grimes, G.R., et al. (2024). Nuclear RNA forms an interconnected network of transcription-dependent and tunable microgels. Preprint at bioRxiv. https://doi.org/10.1101/2024.06.16.599208.
38. Stocks, J., and Gilbert, N. (2024). Nuclear RNA: a transcription-dependent regulator of chromatin structure. Biochem. Soc. Trans. *52*, 1605–1615. https://doi.org/10.1042/BST20230787.
39. Naughton, C., Avlonitis, N., Corless, S., Prendergast, J.G., Mati, I.K., Eijk, P.P., Cockroft, S.L., Bradley, M., Ylstra, B., and Gilbert, N. (2013). Transcription forms and remodels supercoiling domains unfolding large-scale chromatin structures. Nat. Struct. Mol. Biol. *20*, 387–395. https://doi.org/10.1038/nsmb.2509.
40. ENCODE Project Consortium (2012). An integrated encyclopedia of DNA elements in the human genome. Nature *489*, 57–74. https://doi.org/10.1038/nature11247.
41. Corces, M.R., Trevino, A.E., Hamilton, E.G., Greenside, P.G., Sinnott-Armstrong, N.A., Vesuna, S., Satpathy, A.T., Rubin, A.J., Montine, K.S., Wu, B., et al. (2017). An improved ATAC-seq protocol reduces background and enables interrogation of frozen tissues. Nat. Methods *14*, 959–962. https://doi.org/10.1038/nmeth.4396.
42. Stovner, E.B., and Sætrom, P. (2019). epic2 efficiently finds diffuse domains in ChIP-seq data. Bioinformatics *35*, 4392–4393. https://doi.org/10.1093/bioinformatics/btz232.

43. Langmead, B., and Salzberg, S.L. (2012). Fast gapped-read alignment with Bowtie 2. Nat. Methods *9*, 357–359. https://doi.org/10.1038/nmeth.1923.

44. Li, H., Handsaker, B., Wysoker, A., Fennell, T., Ruan, J., Homer, N., Marth, G., Abecasis, G., and Durbin, R.; 1000 Genome Project Data Processing Subgroup (2009). The Sequence Alignment/Map format and SAMtools. Bioinformatics *25*, 2078–2079. https://doi.org/10.1093/bioinformatics/btp352.

45. Zhang, Y., Liu, T., Meyer, C.A., Eeckhoute, J., Johnson, D.S., Bernstein, B.E., Nusbaum, C., Myers, R.M., Brown, M., Li, W., and Liu, X.S. (2008). Model-based Analysis of ChIP-Seq (MACS). Genome Biol. *9*, R137. https://doi.org/10.1186/gb-2008-9-9-r137.

46. Grant, C.E., Bailey, T.L., and Noble, W.S. (2011). FIMO: scanning for occurrences of a given motif. Bioinformatics *27*, 1017–1018. https://doi.org/10.1093/bioinformatics/btr064.

47. Wang, X., and Yue, F. (2023). HiCLift: a fast and efficient tool for converting chromatin interaction data between genome assemblies. Bioinformatics *39*, btad389. https://doi.org/10.1093/bioinformatics/btad389.

48. Abdennur, N., and Mirny, L.A. (2020). Cooler: scalable storage for Hi-C data and other genomically labeled arrays. Bioinformatics *36*, 311–316. https://doi.org/10.1093/bioinformatics/btz540.

49. Plimpton, S. (1995). Fast Parallel Algorithms for Short-Range Molecular-Dynamics. J. Comput. Phys. *117*, 1–19. https://doi.org/10.1006/jcph.1995.1039.

50. Rosa, A., and Everaers, R. (2008). Structure and Dynamics of Interphase Chromosomes. PLoS Comput. Biol. *4*, e1000153. https://doi.org/10.1371/journal.pcbi.1000153.

51. Langowski, J. (2006). Polymer chain models of DNA and chromatin. Eur. Phys. J. E Soft Matter *19*, 241–249. https://doi.org/10.1140/epje/i2005-10067-9.

52. Cook, P.R., and Marenduzzo, D. (2018). Transcription-driven genome organization: a model for chromosome structure and the regulation of gene expression tested through simulations. Nucleic Acids Res. *46*, 9895–9906. https://doi.org/10.1093/nar/gky763.

53. Boyle, S., Flyamer, I.M., Williamson, I., Sengupta, D., Bickmore, W.A., and Illingworth, R.S. (2020). A central role for canonical PRC1 in shaping the 3D nuclear landscape. Genes Dev. *34*, 931–949. https://doi.org/10.1101/gad.336487.120.

54. Strom, A.R., Emelyanov, A.V., Mir, M., Fyodorov, D.V., Darzacq, X., and Karpen, G.H. (2017). Phase separation drives heterochromatin domain formation. Nature *547*, 241–245. https://doi.org/10.1038/nature22989.

55. Brackley, C.A., Liebchen, B., Michieletto, D., Mouvet, F., Cook, P.R., and Marenduzzo, D. (2017). Ephemeral Protein Binding to DNA Shapes Stable Nuclear Bodies and Chromatin Domains. Biophys. J. *112*, 1085–1093. https://doi.org/10.1016/j.bpj.2017.01.025.

56. Hajjoul, H., Mathon, J., Ranchon, H., Goiffon, I., Mozziconacci, J., Albert, B., Carrivain, P., Victor, J.-M., Gadal, O., Bystricky, K., and Bancaud, A. (2013). High-throughput chromatin motion tracking in living yeast reveals the flexibility of the fiber throughout the genome. Genome Res. *23*, 1829–1838. https://doi.org/10.1101/gr.157008.113.

57. Gabriele, M., Brandão, H.B., Grosse-Holz, S., Jha, A., Dailey, G.M., Cattoglio, C., Hsieh, T.-H.S., Mirny, L., Zechner, C., and Hansen, A.S. (2022). Dynamics of CTCF- and cohesin-mediated chromatin looping revealed by live-cell imaging. Science *376*, 496–501. https://doi.org/10.1126/science.abn6583.

58. Fornes, O., Castro-Mondragon, J.A., Khan, A., van der Lee, R., Zhang, X., Richmond, P.A., Modi, B.P., Correard, S., Gheorghe, M., Baranašić, D., et al. (2020). JASPAR 2020: update of the open-access database of transcription factor binding profiles. Nucleic Acids Res. *48*, D87–D92. https://doi.org/10.1093/nar/gkz1001.

59. Imakaev, M., Fudenberg, G., McCord, R.P., Naumova, N., Goloborodko, A., Lajoie, B.R., Dekker, J., and Mirny, L.A. (2012). Iterative correction of Hi-C data reveals hallmarks of chromosome organization. Nat. Methods *9*, 999–1003. https://doi.org/10.1038/nmeth.2148.

60. Finn, E.H., Pegoraro, G., Brandão, H.B., Valton, A.-L., Oomen, M.E., Dekker, J., Mirny, L., and Misteli, T. (2019). Extensive Heterogeneity and Intrinsic Variation in Spatial Genome Organization. Cell *176*, 1502–1515.e10. https://doi.org/10.1016/j.cell.2019.01.020.

61. Cover, T.M., and Thomas, J.A. (2005). Elements of Information Theory (Wiley). https://doi.org/10.1002/047174882X.

STAR★METHODS

KEY RESOURCES TABLE

REAGENT or RESOURCE	SOURCE	IDENTIFIER
Deposited data		
H3K27ac ChIP-seq	ENCODE[40]	ENCFF197QHX; ENCFF882PRP
H3K27me3 ChIP-seq	ENCODE[40]	ENCFF622QTG; ENCFF458YDD
H3K9me3 ChIP-seq	ENCODE[40]	ENCFF331ODM; ENCFF782FRS; ENCFF138CTR
Control ChIP-seq for all histone modifications	ENCODE[40]	ENCFF232FPZ; ENCFF624TPS; ENCFF339YTX; ENCFF430BAQ
CTCF ChIP-seq	ENCODE[40]	ENCFF389RQC; ENCFF343LTM; ENCFF232FPZ; ENCFF624TPS
Rad21 ChIP-seq	ENCODE[40]	ENCFF311CJK; ENCFF800DLO; ENCFF963CVB
ATAC-seq	Corces et al.[41]	SRR5427886; SRR5427887
Hi-C	Rao et al.[15]	4DNFIYECESRC
Micro-C	Krietenstein et al.[24]	4DNFINYO612N
3D structures of individual gene topos	This paper	https://3dgene.igc.ed.ac.uk/
Processed simulation and experimental data for generating the figures; example scripts for running the simulation model using LAMMPS	This paper	https://doi.org/10.7488/ds/7821
Software and algorithms		
Epic2 (v.0.0.52)	Stovner and Sætrom[42]	https://github.com/biocore-ntnu/epic2
Bowtie2	Langmead and Salzberg[43]	https://github.com/BenLangmead/bowtie2
SAMtools (v1.19)	Li et al.[44]	https://github.com/samtools/samtools
Picard tools (v2.21.9)	Broad Institute	http://broadinstitute.github.io/picard
MACS2 (v2.2.7.1)	Zhang et al.[45]	https://github.com/macs3-project/MACS
FIMO	Grant et al.[46]	https://meme-suite.org/meme/doc/fimo.html
HiCLift (v1.0)	Wang and Yue[47]	https://github.com/XiaoTaoWang/HiCLift
Cooler (v0.9.3)	Abdennur and Mirny[48]	https://github.com/open2c/cooler
LAMMPS (version 12Dec2018)	Plimpton[49]	https://github.com/lammps/lammps

METHOD DETAILS

Polymer modeling

The e-HiP-HoP framework involves coarse-grained molecular dynamics simulations, in which collections of molecules are represented by beads, which interact with phenomenological force fields and move according to Newton's laws.[8] More specifically, chromatin fibers and chromosomes were modeled as bead-and-spring polymers, while complexes of transcription factors (TFs) or other chromatin-binding proteins were represented by additional individual beads. We also included loop extruding factors, represented by additional springs between non-adjacent chromatin chain beads, and chromatin heteromorphism. We used the multi-purpose molecular dynamics package Large-scale Atomic/Molecular Massively Parallel Simulator (LAMMPS).[49] Typically, each simulation was run with between 8 and 16 cores and required approximately 800 computational core hours. In this section, we detail the potentials underlying the force fields used in the simulations.

The chromatin fiber

A chromatin fiber corresponding to a whole chromosome, or chromosome fragment, was discretized as a set of monomers, each of size corresponding to 1 kbp, or to $\sigma \sim 20-30$ nm, which can be determined by fitting to microscopy experiments (see below). This resolution is comparable to that used in related coarse-grained models for chromatin.[14,50] Any two monomers (i and j) in the chromatin fiber interact purely repulsively via the Weeks-Chandler-Andersen (WCA) potential, given by

$$U_{\mathrm{WCA}}(r_{ij}) = \begin{cases} 4k_BT\left[\left(\frac{\sigma}{r_{ij}}\right)^{12} - \left(\frac{\sigma}{r_{ij}}\right)^{6} + \frac{1}{4}\right] & \text{if } r_{ij} < 2^{1/6}\sigma \\ 0 \text{ otherwise,} & \end{cases} \quad \text{(Equation 1)}$$

where r_{ij} is the separation of beads *i* and *j*. There is also a finite extensible non-linear elastic (FENE) spring acting between consecutive beads in the chain to enforce chain connectivity. This is given by

$$U_{\mathrm{FENE}}(r_{ij}) = -\frac{K_f R_0^2}{2}\ln\left[1-\left(\frac{r_{ij}}{R_0}\right)^2\right], \quad \text{(Equation 2)}$$

where *i* and *j* are neighboring beads, $R_0 = 1.6\sigma$ is the maximum separation between the beads, and $K_f = 30k_BT/\sigma^2$ is the spring constant. Additionally, a triplet of neighboring beads in the chromatin fiber interact via a Kratky-Porod term to model the stiffness of the chromatin fiber, which explicitly reads as follows:

$$U_{\mathrm{KP}}(\theta_{ij}) = \frac{k_BTl_p}{\sigma}(1-\cos\theta_{ij}), \quad \text{(Equation 3)}$$

where *i* and *j* are neighboring beads, while θ_{ij} denotes the angle between the vector connecting beads *i* and *j* = *i+1* and the vector connecting beads *j* and *j+1*. The quantity l_p is related to the persistent length of the chain: this parameter was set to 4σ in our simulations, corresponding to a relatively flexible chain, and in line with the values of persistence length used for coarse-grained modeling of chromatin at resolution similar to the one chosen here of 1 kbp per bead.[14,50,51] Note, however, that the effective persistence length of the polymer can differ from the parameter value, as it depends on the local chromatin environment and its heteromorphic compaction. Its effective value is also scale dependent and will be significantly different from the bare value set here when looking at conformations of chromatin over hundreds of kbp or Mbp, as these will typically include large inactive, or heterochromatic, regions.

Multivalent chromatin-binding proteins

Multivalent TFs were modeled as spheres, again with size σ for simplicity. The interaction between a chromatin bead, *a*, and a multivalent TF, *b*, was modeled via a truncated and shifted Lennard-Jones potential, given by

$$U_{\mathrm{LJ/cut}}(d_{ab}) = \begin{cases} \dfrac{4\epsilon_{ab}}{\mathcal{N}}\left[\left(\dfrac{\sigma}{d_{ab}}\right)^{12}-\left(\dfrac{\sigma}{d_{ab}}\right)^{6}-\left(\dfrac{\sigma}{r_c}\right)^{12}+\left(\dfrac{\sigma}{r_c}\right)^{6}\right] & \text{if } d_{ab}<r_c \\ 0 \text{ otherwise,} \end{cases} \quad \text{(Equation 4)}$$

where d_{ab} denotes the distance between the centers of the chromatin and protein beads, $r_c = 1.8\sigma$ is a cut-off parameter, and $\mathcal{N}$ is a normalization constant that ensures the depth of the potential reaches $-\epsilon_{ab}$ at the minimum point.

Three species of multivalent TFs were considered: a generic active TF and two inactive TFs, one modeling polycomb-like proteins and another modeling heterochromatin protein 1 (HP1)-like, or other heterochromatic, proteins. Active TFs bind strongly ($\epsilon_{ab} = 7k_BT$) to beads with high accessibility (corresponding to ATAC peaks; for more details on this and on chromatin bead coloring, see ***Simulation input data***) and weakly ($\epsilon_{ab} = 3k_BT$) to those enriched in H3K27ac. This aims to capture promoter-enhancer interactions and the formation of transcriptional domains.[52] For inactive TFs, polycomb-like TFs bind to beads enriched in H3K27me3 ($\epsilon_{ab} = 7k_BT$), representing interactions mediated by polycomb repressive complexes[53] (PRCs), while heterochromatic TFs bind to beads with H3K9me3 ($\epsilon_{ab} = 3k_BT$), modeling bridging facilitated by HP1.[54] Importantly, TFs only interact with one another via steric repulsion (described by the WCA potential, Equation 1). Nevertheless, thanks to their ability to bridge between multiple chromatin beads with similar marks, TFs of different species tend to microphase separate and form individual clusters via the bridging-induced attraction[13] (Figure 1B). In addition to TF-chromatin binding, there is also a weak, direct chromatin-chromatin interaction ($\epsilon_{ab} = 0.4k_BT$) between beads without the active H3K27ac mark. This extra ingredient facilitates the phase separation between euchromatin and heterochromatin.

TFs can switch back and forward between a binding and a non-binding state with a rate k_{sw}. This feature mimics post-translational modifications on protein complexes and accounts for the dynamical turnover of constituents within nuclear protein clusters.[55] When TFs are non-binding, they interact with chromatin beads via steric repulsion (modeled by the WCA potential).

Loop extrusion

Loop extruding factors, representing for instance the structural maintenance of chromosome (SMC) complex cohesin, were incorporated in the e-HiP-HoP framework in a way similar to previous work.[14] Each loop extruder was described as a dimer whose two ends move divergently along the fiber. For simplicity, this process was modeled implicitly as a harmonic spring with short range WCA repulsion between the chromatin beads *i* and *j* to which the two ends of an extruder are instantaneously bound,

$$U_{\mathrm{extr}}(r_{ij}) = U_{\mathrm{WCA}}(r_{ij}) + k_{\mathrm{extr}}(r_{ij}-r_0)^2, \quad \text{(Equation 5)}$$

where $k_{\mathrm{extr}} = 40k_BT/\sigma^2$ is the harmonic spring constant and $r_0 = 1.5\sigma$ the harmonic bond length. In the simulations, an extruder could bind randomly to any chromatin bead, say the *i*-th one, and this was modeled by introducing a spring linking beads *i* and *i+3* (as a crumpled spring may already connect beads *i* and *i+2* to model the heteromorphic fiber, see below). Once bound, the two ends of an extruder were assumed to translocate at speed v_{extr}, and this was done by moving the spring to the next pair of beads. Both ends of

an extruder move along the fiber until colliding another extruder or reaching a CTCF bead (see below) with opposite orientation to the direction of travel. Note that the two ends move independently: if one end halts due to the aforementioned scenarios, the other can continue to extrude. Extruders detach from chromatin with rate k_{off}, upon which the spring is removed. For simplicity, it was assumed that when an extruder unbinds from the fiber, another immediately re-attaches elsewhere (i.e., the number of extruders on chromatin remains constant). The loop extrusion dynamics was performed using a python script which drives the LAMMPS library.

Heteromorphic chromatin modeling

A prominent feature of the e-HiP-HoP framework is that it represents chromatin as a heteromorphic polymer, whose linear compaction varies along the contour. This feature is needed to account for a more open and disrupted conformation in acetylated regions, as observed in FISH experiments.[12] In practice, the heteromorphic property was implemented by introducing extra harmonic springs which link next-to-nearest neighbor beads along the chain, i and $i+2$, in regions that are not annotated with the H3K27ac mark. Here, the spring constant was set to $k_h = 200k_BT/\sigma^2$ and the bond length to $r_0 = 1.1\sigma$. In this way, regions with the extra springs become crumpled and have higher linear compaction, whereas those with the mark remain open. This variability in local folding changes the stiffness and persistence length of the fiber.[12]

Equations of motion

The time evolution of each bead i in the simulations (whether TF or chromatin bead) is governed by the following Langevin equation:

$$m_i\frac{d^2r_{i,\alpha}}{dt^2} = -\nabla_{i,\alpha}U_i - \gamma_i\frac{dr_{i,\alpha}}{dt} + \sqrt{2k_BT\gamma_i}\,\eta_{i,\alpha}, \qquad \text{(Equation 6)}$$

where α denotes Cartesian vector components, $r_{i,\alpha}$ is the α-th component of the position vector of bead i, U_i is the total potential experienced by it, m_i and γ_i are its mass and friction coefficients (these coefficients are the same for all beads in our simulations, denoted by m and γ), and $\eta_{i,\alpha}$ is the α-th component of a stochastic noise vector with the following mean and variance:

$$\langle\eta_{i,\alpha}\rangle = 0,\ \langle\eta_{i,\alpha}(t)\eta_{j,\beta}(t')\rangle = \delta_{ij}\delta_{\alpha\beta}\delta(t-t'), \qquad \text{(Equation 7)}$$

where the Latin and Greek indices run over beads and Cartesian components, respectively, and the first two δ's denote Kronecker deltas, while the last one a Dirac delta.

Simulation units and parameters

Our basic unit of length is the chromatin bead size σ, which sets the resolution and equals 1 kbp. Since the packaging of DNA into chromatin is not fully understood, we did not fix the physical value of the length unit in nm ahead of running the simulations. Instead, we performed the simulations and estimated the physical value of σ by comparing with experimental data, typically FISH (see below).

Regarding timescales, we note that the previously defined simulation units give rise a natural time unit, $\tau = \sqrt{\sigma^2 m/(k_BT)}$. Another important timescale is the Brownian time $\tau_B = \sigma^2/D$, with $D = k_BT/\gamma$ the diffusion coefficient of the beads. The Brownian time gives an order-of-estimate measure of the time it takes for a bead to diffuse across its own diameter, and it is this timescale which we use to determine the mapping of simulation time to real time. In simulation units, for simplicity we work with all beads having the same mass $m = 1$ and set $\gamma = 2$, so that $\tau_B = 2\tau$. This means that the system is overdamped, as should be the case physically; however, beads have more inertia than in reality (this is necessary to keep overall simulation times manageable), but this only affects the dynamics at very early times, whereas we are interested in chromatin conformations at steady state. To map between simulation and real time, we measured the average mean squared displacement for all polymer beads and found a value of τ_B which gives a good fit to experimental results from ref. [56], corresponding to $\tau_B \sim 8$ ms [assuming a bead size $\sigma \sim 25$ nm, which is within the range inferred from fitting to FISH data (Figures 2 and S4)]. Numerical integration of Equation 6 was performed using a standard velocity-Verlet algorithm with time step $dt = 0.01\tau$, implemented in the LAMMPS engine.[49]

Parameter values used in the simulations and not specified in the ***Polymer modeling*** section above were chosen as follows. The number of TF beads was set to 10% of the number of chromatin beads, while the ratio of active, polycomb-like, and heterochromatic TFs was fixed at 1/4:1/8:5/8. Additionally, the switching rate was set to $k_{\text{sw}} = 10^{-3}\tau^{-1}$. As for loop extrusion, the number of extruders bound to chromatin was fixed to give a density of 10 extruders/Mbp. Extrusion and unbinding rates were set to $v_{\text{extr}} = 4\times10^{-3}$ kbp$/\tau$ and $k_{\text{off}} = 2.5\times10^{-5}\tau^{-1}$, respectively. While these parameters were chosen to facilitate sampling and are less realistic individually, the ratio $\lambda = v_{\text{extr}}/k_{\text{off}} = 160$ kbp, also known as the extruder's processivity, and the density of extruders (or the average spacing between them) are all in line with values used in the literature.[14] We note that the slightly smaller optimized density of extruders of $\sim$4 extruders/Mbp found in ref. [57] applies to mouse, so it is not directly transferrable to the human chromatin case.

The chromatin fibre was simulated within a periodic cube of length L such that the density of chromatin is $\sim$6.5 Mbp/μm^3. This is based on the fact that there are around 6.5 Gbp of DNA in a human diploid cell, and that the diameter of a typical cell nucleus is $\sim$10 μm.

To facilitate computation, chromosomes were simulated individually rather than as a whole (Figure S1). While this approach neglects long-range and interchromosomal (or *trans*) interactions, this is acceptable in our case as the main objective of the work is to examine the local chromatin structure and *cis* interactions of regulatory elements, which typically span no longer than a few Mbps. In practice, shorter chromosomes (HSA14, 15, and 17 to 22) were simulated as a single segment, whereas longer

chromosomes (HSA1 to 13, 16, and X) were each divided into smaller fragments. Breakpoints were chosen to be sites where there is low enrichment in chromatin modifications within their neighborhood (i.e., gene deserts; see Figure S1).

Initialization and simulation protocol

The simulation system was initialized as follows. As described in ref. 12,[50], the chromatin fiber was first generated as a mitotic-like helix conformation – i.e., a stack of rosettes (see ref. [50] for details on the equations used to create these). The initial simulation box was a parallelepiped, with equal smaller dimensions in the x and y directions and a longer height that depended on the length of the initially cylindrical chromosome fragment along z. To relax the fiber from the rosette conformation, beads along the chain were initially connected together using harmonic springs (with spring constant equal to $100 k_B T/\sigma^2$ and bond length equal to 1.1σ), and all non-neighbor pairwise interactions were governed by the repulsive soft potential (whose strength gradually increased from 0 to $500 k_B T$). After an initial relaxation simulation of duration 600τ, the springs were replaced by FENE bonds, and the soft potential by the WCA potential. The simulation box was then compressed slowly along the z direction for $10^4\tau$ such that it became a cube with the desired volume. Next, the fiber was relaxed further within the cube with fixed boundaries for $5 \times 10^3\tau$ and then with periodic boundaries for $2.5 \times 10^4\tau$. To allow the fiber to quickly lose memory of the rosette-like conformation, extruders (with a density of $\sim$7.5 extruders/Mbp) were loaded to the fiber to perform loop extrusion without any CTCF boundaries for $2 \times 10^4\tau$. Harmonic springs for modeling fiber heteromorphism were then added to the entire fiber, and the fiber was allowed to relax for $10^4\tau$. Next, TF beads were incorporated and allowed to equilibrate with the fiber for $10^3\tau$, with TF-chromatin interactions being purely repulsive (modeled by the WCA potential). Finally, regions enriched in H3K27ac had their crumple springs removed such that the fiber had different levels of local compaction.

Ten independent runs were conducted using this procedure for each chromosome fragment, and their final conformations were used as the starting conditions for 300 production runs (which were performed using different random seeds). From measuring structural properties such as the radius of gyration of the fiber, it was verified that production runs starting from the same relaxed conformation gave different structures. In the production run, the chromatin fiber was simulated for a period of $3 \times 10^5\tau$. Attractive chromatin-chromatin interactions were switched on at time $10^4\tau$, and subsequently TF-chromatin interactions at $5 \times 10^4\tau$. The system was sampled (e.g., for contact map calculation) every $2 \times 10^3\tau$ during the final $10^5\tau$ of the simulation period. The 600 structures shown in the main text were generated at times $2 \times 10^5\tau$ and $3 \times 10^5\tau$; the choice of how many structures to retain was motivated by the requirement for structures chosen to be statistically uncorrelated.

Simulation input data

Chromatin beads were assigned different states or colors according to their local epigenetic modifications and DNA accessibility. Three epigenetic modifications were used here: H3K27ac, H3K27me3, and H3K9me3. These marks are typically associated with actively transcribed euchromatin, facultative heterochromatin, and constitutive heterochromatin, respectively. Their ChIP-seq profiles (for GM12878 cells) were obtained from the Encyclopedia of DNA Elements (ENCODE) database[40] (https://www.encodeproject.org). Specifically, BAM files aligned to the hg19 reference genome were downloaded, both for the "signal" and the "control" experiments (see key resources table). Peaks were then called using the Epic2 software.[42] H3K27ac peaks were used to determine disrupted regions of the chromatin fiber, where no additional crumpling springs were applied; H3K27me3 and H3K9me3 peaks were used to set binding sites for polycomb-like and heterochromatic proteins, respectively.

DNA accessibility was determined from an ATAC-seq dataset.[41] ATAC-seq peaks were used to identify high-affinity binding sites for multivalent active proteins. To determine the peaks, raw FASTQ files (see key resources table) were downloaded and aligned to hg19 using Bowtie2.[43] Mitochondrial reads and PCR duplicates were filtered out using SAMtools[44] and Picard tools, and low-quality mapped reads (MAPQ < 30) were also removed. Peaks were then called using the software Model-based Analysis of ChIP-seq 2 (MACS2)[45] and were further filtered to remove those with a low fold-enrichment signal.

Chromatin beads were assigned particular epigenetic and/or ATAC marks if the respective chromatin regions have significant enrichment, or peaks, of these marks. Note that a bead could have more than one kind of mark, and its properties were determined based on the combination of marks it possesses.

To identify the CTCF binding sites which constrain loop extrusion, ChIP-seq datasets for CTCF and RAD21, a subunit of the cohesin complex, were obtained from ENCODE. BAM files aligned to hg19 for both datasets (see key resources table) were downloaded, and peaks were called using MACS2. For each CTCF peak, we determined the binding orientation by locating within the peak the underlying sequence for the CTCF binding motif (obtained from JASPAR[58]) using the program Find Individual Motif Occurrences (FIMO).[46] If multiple motifs with different directions were found, we used the motif that matches most closely to the consensus (if motifs with opposite directions scored similarly when compared to the consensus, we labeled the peak as bidirectional).

Genomic loci which had peaks in both CTCF and RAD21 ChIP-seq profiles while also containing the CTCF binding motif were marked as candidate binding sites. Beads covering these sites were then labeled as CTCF beads, and the direction in which they act on the extruders was based on the orientation of the underlying motif. Motivated by the cell-to-cell variability in CTCF binding, CTCF beads were activated stochastically in each simulation run according to a probability that was linearly proportional to the score of the corresponding CTCF peak, similarly to ref. 12. When there were multiple peaks within the same bead, all possible outcomes were considered. For example, if a bead encompassed both a forward and a backward-oriented CTCF binding site, the probabilities

of the bead being a forward, backward, bidirectional or inactive CTCF boundary were calculated based on the score of individual peaks, and an outcome was selected based on these probabilities.

Comparison with Hi-C, Micro-C, and FISH experiments

Before e-HiP-HoP could be used genome wide in a predictive way, it was important to validate it by showing that it gives results consistent with experimental data. To do so, e-HiP-HoP predictions were compared to Hi-C, Micro-C, and FISH. Below we describe each of these comparisons in turn.

Comparison of contact maps between simulations, Hi-C, and Micro-C

To assess the ability of e-HiP-HoP simulations to predict structural data, contact maps were generated from simulations and compared to those from Hi-C[15] and Micro-C experiments.[24] PAIRS files from both sets of experiments were downloaded from the 4DNucleome Data Portal (see key resources table). To facilitate comparison with simulation data, reads were re-aligned to hg19 using HiCLift.[47] Contact maps were then generated using Cooler,[48] at 1 kbp resolution for Micro-C and 10 kbp resolution for Hi-C, and they were normalized using the iterative correction and eigenvector decomposition (ICE) strategy.[59] Simulated maps were constructed in a way inspired by the cross-linking step in Hi-C for sampling pairwise chromatin interactions.[12] As previously mentioned, the conformation of the chromatin fiber was sampled every $2\times10^3\tau$ over a period of $10^5\tau$. For each sampled structure, the following procedure was conducted. First, a pair of chromatin beads, say i and j, were selected randomly with their separation r_{ij} computed. Then, the pair was accepted and registered as a "read" in the contact matrix with probability

$$p_{\text{read}}(r_{ij}) = \exp\left(-\frac{r_{ij}}{r_t}\right), \qquad \text{(Equation 8)}$$

where r_t is a distance threshold (set to 3.5σ for comparison with Hi-C and 1.5σ for comparison with Micro-C) related to the separation within which crosslinking is effective. By repeating this procedure many times and over different structures and simulation runs, reads were piled up in a way similar to that in Hi-C and Micro-C. To aid visual and quantitative comparison between simulated and experimental contact maps, we fixed the total number of reads in a simulated map to be of the same order as that in a Hi-C or Micro-C map.

We considered two different approaches to quantify the similarity between simulated and experimental contact maps. First, we computed the Pearson correlation coefficient from correlating the maps directly, and the results are shown above each map in Figures 2 and S3. Second, since the interaction probability between two loci is strongly dependent on their genomic distance, which can impact the direct correlation score, we also considered a metric that is less "distance-sensitive". In particular, we compared the patterns of topologically associating domains (TADs) seen in both simulated and Hi-C maps by computing the directionality score $D(i)$, which detects TADs algorithmically using the fact that near their boundaries, interactions are highly favored toward either the left or right of a chromatin segment (Figure S3C). The D score captures this bias and is defined for each chromatin bin (say bin i) in the map by subtracting the interactions in the region to the left of i by the interactions to the right of i. For both right and left interactions, contacts were summed up between a lower and an upper end threshold. The lower end threshold was fixed at 20 kbp to avoid artifacts close to the diagonal within the Hi-C map, whereas the upper end threshold was set to 500 kbp (varying between 100 kbp and 2 Mbp showed no significant differences). Note that because this score compares the aggregated interactions within regions either side of a bin up to the same genomic distance away, the distance dependence is effectively canceled out. The similarity between the simulated map and the Hi-C map can then be determined based on the Pearson correlation of $D(i)$ in both maps (Figures S3C–G).

Comparison with FISH experiments

Another way to compare predicted and observed 3D chromatin structure is to simulate FISH experiments. To do so, three pairs of FISH probes were considered from experiments using SATO3 lymphoblastoid cells[23] (Figures 2B and 2C), together with six pairs of probes from experiments using the human retinal pigment epithelial 1 (RPE1) cells[27] (Figure S4). For each pair, to generate probe separation measurements from our simulation data, we identified chromatin beads associated with each probe and took its position to be the center of mass of these beads. By measuring the separation of the centers of mass of beads mapping to the pair of probes in each of the 600 simulated structures, a distribution of separations, in units of σ, was constructed.

This simulated distribution was compared to the corresponding experimental one. To perform this comparison quantitatively, we mapped simulation length units to physical ones. For each pair, we used the two-sample Kolmogorov-Smirnov (KS) statistics to determine the distance between the simulated distribution and the corresponding experimental one and found the value of σ in nm which minimized this distance. We then computed the p value corresponding to the probability that the experimental and simulated distributions were the same. This procedure identified that experimental and simulated distributions were not different in a statistically significant way ($p > 0.1$) in all but one case (see Figure S4). This case corresponded to a gene-poor region (1p31.2), which may be less accurately represented by our simulations. Additionally, experimental measurements in this case were based on RPE1 cells, which may have a different local environment in that region with respect to the GM12878 lymphoblastoid cells that we simulated. To facilitate comparison between simulated and experimental distributions, in both Figures 2C and S4 we normalized the distributions by the median of the experimental distribution for each probe.

Analysis of 3D simulated chromatin structure

Within the main text, we discussed the chromatin interaction networks associated with a gene topos, and these networks were identified as follows. We first defined partners of a promoter as ATAC beads which contact (i.e., separated by less than 3.5σ) that promoter in over 10% of the simulated structures, and we then defined a gene topos as the union set of a promoter and all its partners. Given these definitions, we determined the interaction networks within a topos, or the different ways that the promoter of interest interacts with its partners (Figure 5A). Figure 5B in the main text displays a plot of the number of networks against the number of partners for all gene topoi, showing that the former quantity increases rapidly with the latter, until saturating due to the limited number of conformations. This increase, to a first approximation, can be understood from a simple combinatorial calculation. If one neglects the polymeric nature of chromatin, finding the number of unique networks for q partners is equivalent to counting the number of ways to create a subset within a set of q (distinguishable) elements. As each element can either be in the subset or not, this number is simply 2^q. Clearly, this constitutes the maximum possible number of networks, as physical constraints of the fiber would render some unachievable. Additionally, it is not clear *a priori* whether all the remaining permissible networks would occur in reality, as other factors, such as the local chromatin context and linear spacing between ATAC sites, can also influence the networks. Nevertheless, Figure 5B shows that even with a modest number of partners, many networks were detected for individual gene topoi, suggesting that these topoi display large heterogeneity in their 3D folding across the ensemble of simulated structures. This result is consistent with previous single-cell studies revealing the extensive variation in chromatin organization within a population of cells.[60]

Shannon entropy and structural diversity

The *SMARCA5* and *GINS4* topoi discussed as examples in Figures 5D and 5E demonstrate that the number of conformations associated with individual networks can vary substantially. In *SMARCA5*, many conformations are mapped to a single network (Figure 5D), whereas in *GINS4*, they are spread more evenly across a larger number of networks (Figure 5E). We quantified how the population of structures is distributed among the observed networks by a structural diversity score H, which is inspired by the Shannon diversity index or Shannon entropy S.[61] Specifically, we defined

$$H = \frac{S}{q+1} = \frac{1}{q+1}\sum_{i=1}^{N} - n_i \ln n_i, \quad \text{(Equation 9)}$$

where q is the number of partners, n_i is the fraction of structures in network i, and N denotes the total number of networks. We incorporated the normalization factor $1/(q+1)$ as S is expected to scale linearly with q (the addition of one in the denominator ensures that H remains well-defined in the case where a gene has no partners). This can be seen, for instance, from maximizing S, which is achieved when $n_i = 1/N$. In this case, $S = \ln N$, and substituting the upper bound $N = 2^q$ gives $S = q \ln 2$. For this reason, the most informative genome-wide correlations described in the result section were found with normalized structural diversity [i.e., $S/(q+1)$].

QUANTIFICATION AND STATISTICAL ANALYSIS

All statistical details are indicated in the STAR Methods, results, or figure legends.

Cell Genomics, Volume *4*

Supplemental information

Genome-wide chromosome architecture prediction reveals biophysical principles underlying gene structure

Michael Chiang, Chris A. Brackley, Catherine Naughton, Ryu-Suke Nozawa, Cleis Battaglia, Davide Marenduzzo, and Nick Gilbert

SUPPLEMENTAL FIGURES

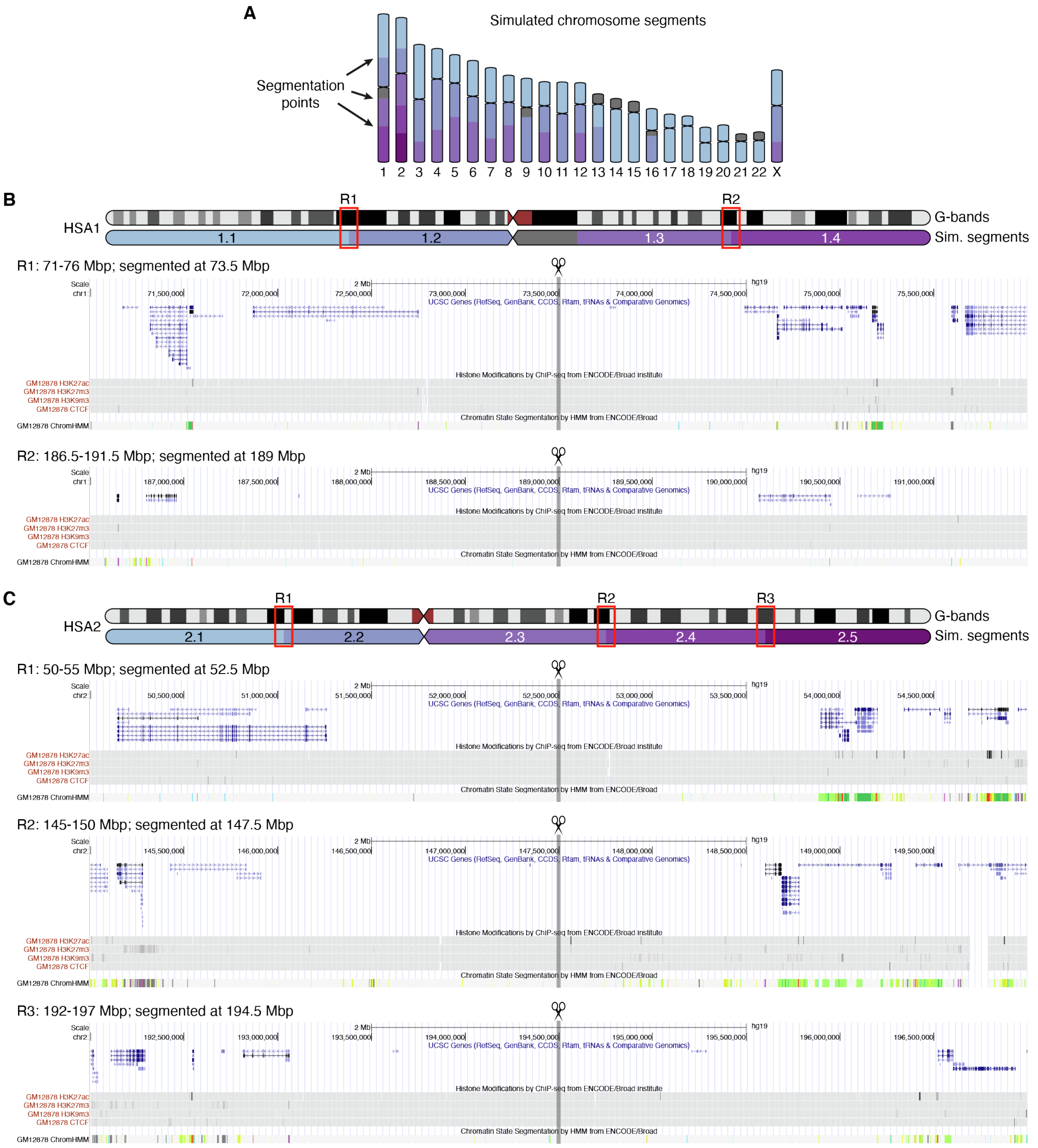

Figure S1. Long chromosomes were divided into shorter fragments when performing e-HiP-HoP simulations, related to Figure 1 and STAR Methods.

(A) Ideograms showing the segmentation points within each chromosome.

(B and C) Simulated chromosome fragments in (B) HSA1 and (C) HSA2 and the chromatin context near segmentation points. Ideograms at the top indicate the positions of segmentation (the region colored in gray is unmappable within the hg19 reference genome and was not simulated). Snapshots from the UCSC genome browser (bottom) show the genes, various ChIP-seq signals (H3K27ac, H3K27me3, H3K9me3, and CTCF), and the 15 chromatin-state hidden Markov model (ChromHMM) track within a 5 Mbp region around segmentation points. These tracks demonstrate that there are no notable genomic and epigenomic features near the segmentation points.

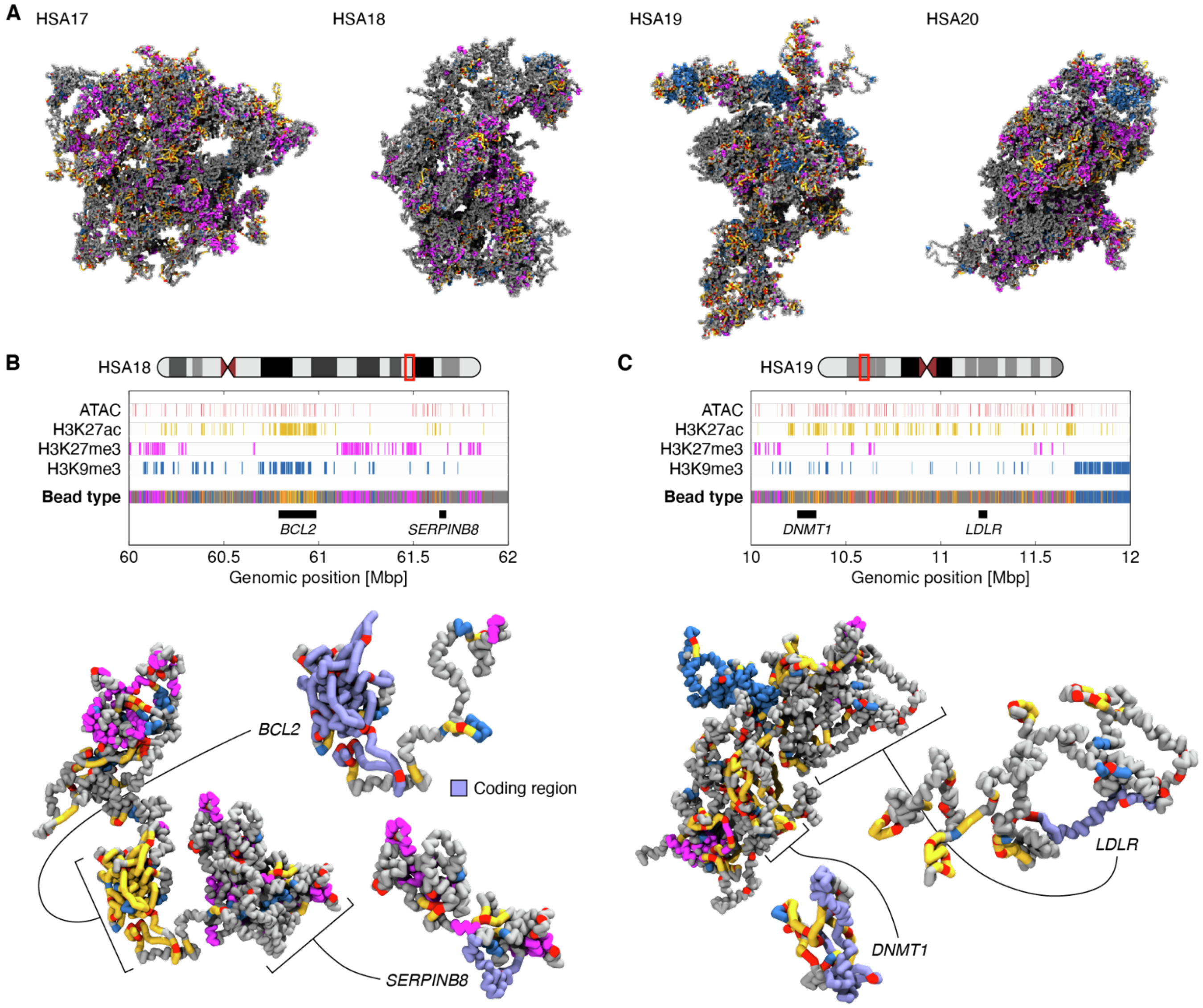

Figure S2. Simulated structures from e-HiP-HoP provide a detailed view of the 3D conformation of individual genes and their neighborhood, related to Figure 1.

(A) Representative snapshots of the predicted structures of HSA17, 18, 19, and 20, all of which were simulated individually as a single, continuous polymer without segmentation.

(B) An enlarged view of HSA18 at 60–62 Mbp. Epigenetic tracks used as model inputs and the resulting bead type along the chromatin fiber are shown at the top, and an example structure of this region is shown at the bottom, with a further expanded view of two gene topoi, *BCL2* and *SERPINB8* (their coding region is colored in light purple).

(C) Similar to (B) but for the region 10–12 Mbp in HSA19, with the conformations of the topoi of genes *DNMT1* and *LDLR* shown.

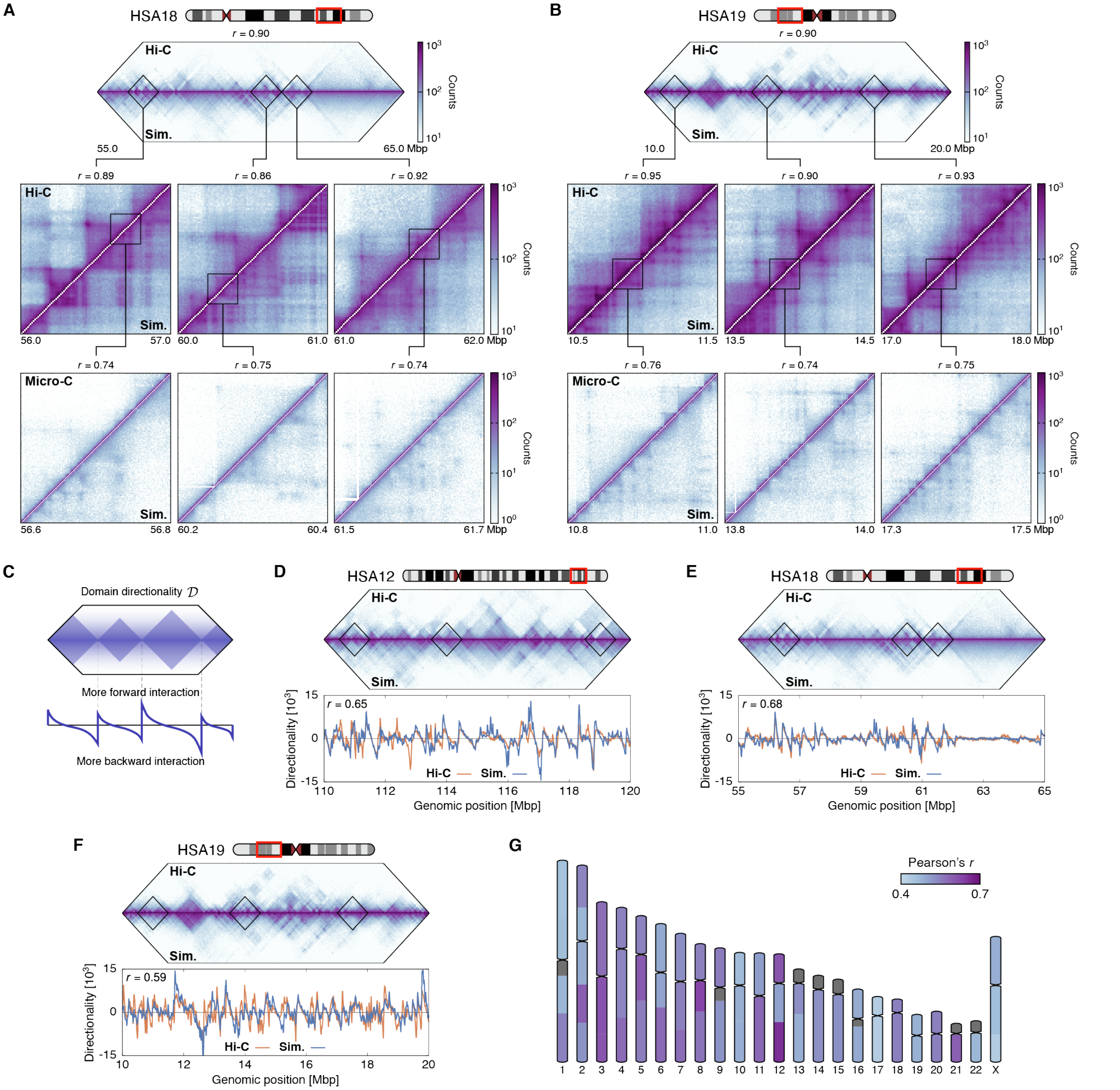

Figure S3. Comparison of contact maps between Hi-C, Micro-C, and e-HiP-HoP simulations, related to Figure 2.

(A and B) Comparison of contact maps between Hi-C, Micro-C, and e-HiP-HoP simulations for the regions (A) HSA18:55–65 Mbp and (B) HSA19:10–20 Mbp. The top panel shows the comparison of the full region between Hi-C and simulations at 10 kbp resolution, with enlarged views of the 1 Mbp boxed regions shown in the middle panel. The bottom panel compares the maps between Micro-C and simulations at 1 kbp resolution for the 200 kbp boxed regions marked in the middle panel. Pearson correlation coefficient *r* is reported for each comparison, and all correlations are statistically significant with $p < 10^{-10}$.

(C) A schematic explaining the directionality score *D* which quantitatively captures domains seen in contact maps. The *D* score at a chromatin bin is the total contact frequencies of the bin with other bins to its left, up to a threshold, minus the total contact frequencies with those to its right (see STAR Methods).

(D–F) Comparing the directionality profiles of the contact maps between Hi-C and simulations for (D) the region HSA12:110–120 Mbp (as examined in Figure 2A) and the two regions shown above, (E)

HSA18:55–65 Mbp and (F) HSA19:10–20 Mbp. Here, the top panel shows the comparison of the contact maps at 10 kbp resolution, and the bottom panel plots the directionality profiles. Pearson correlation coefficient *r* is reported for comparing these profiles and is statistically significant ($p < 10^{-10}$) for all the regions shown here.

(G) Ideograms showing the Pearson correlation coefficient between the directionality profiles from Hi-C and simulated contact maps for each chromosome segment (gray regions were not simulated as they are unmappable regions within the reference genome).

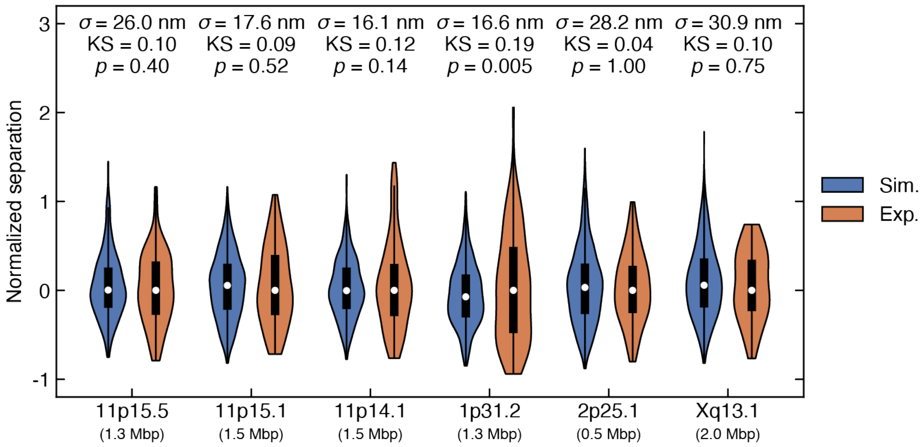

Figure S4. Further validation of e-HiP-HoP simulations with FISH data, related to Figure 2.
Comparing the simulated and experimental FISH distance distributions for six pairs of probes. As in Figure 2C, we tested whether the difference between the distributions is statistically significant using the two-sample Kolmogorov-Smirnov (KS) test (see STAR Methods). Of all, the simulated and experimental distributions only show significant deviation for the probe pair within 1p31.2, which is a gene-poor region and is therefore less relevant to our analysis on actively transcribed chromatin. Experimental data were taken from ref. S1.

Supplemental References

[S1] Nozawa, R.S., Boteva, L., Soares, D.C., Naughton, C., Dun, A.R., Buckle, A., Ramsahoye, B., Bruton, P.C., Saleeb, R.S., Arnedo, M., et al. (2017). SAF-A Regulates Interphase Chromosome Structure through Oligomerization with Chromatin-Associated RNAs. Cell *169*, 1214–1227.e18. https://doi.org/10.1016/j.cell.2017.05.029.
